# Empathic disequilibrium as a new framework for understanding individual differences in psychopathology

**DOI:** 10.3389/fpsyg.2023.1153447

**Published:** 2023-05-19

**Authors:** Ido Shalev, Alal Eran, Florina Uzefovsky

**Affiliations:** ^1^Psychology Department, Ben Gurion University, Beer-Sheba, Israel; ^2^Computational Health Informatics Program, Boston Children's Hospital, Boston, MA, United States

**Keywords:** empathy, cognitive empathy, emotional empathy, psychopathology, social cognition

## Abstract

**Introduction:**

Empathy is part of basic social cognition and is central to everyday interactions. Indeed, emotional and cognitive empathy deficits are related to various psychopathologies, yet the links reported have been inconsistent. Thus, the mechanism underlying these inconsistent links is poorly understood. At least a partial answer may lie in that the dependency between cognitive and emotional empathy has been overlooked. Here, we examined the (dis)equilibrium between emotional and cognitive empathy and how it relates to individual differences in clinical traits. We further examined a possible mediator of these links—emotional reactivity.

**Methods:**

Participants (*N* = 425) from the general population reported on their empathy, emotional reactivity, autistic traits, psychopathic tendencies, and symptoms of depression and anxiety.

**Results:**

Beyond empathy, both extremes of empathic disequilibrium were associated with various features of clinical conditions; Higher emotional relative to cognitive empathy was related to the social domain of autism and anxiety, while higher cognitive relative to emotional empathy was related to the non-social domain of autism, depression symptoms, and psychopathic tendencies. The associations with autistic traits, anxiety, and psychopathic tendencies were mediated by emotional reactivity.

**Discussion:**

Our findings suggest a new framework for understanding how individual variability in empathy is expressed in various psychopathologies.

## Introduction

Our fundamental ability to empathize, namely, of understanding and sharing emotions, is the cornerstone of socializing and maintaining interpersonal relationships. While it is clear that impairments in empathy are related to a wide range of psychological conditions, across the typical to the clinical range, the reason for such links remains mostly unclear (Gonzalez-Liencres et al., 2013). While most relevant research focuses on the relationship between empathy and a single psychopathology or psychopathological tendency (in the typical range), neurobehavioral and genetic findings suggest that focusing on a transdiagnostic approach, i.e., examining the underlying relationship across dimension of psychopathology, may lead to a clearer understanding of human traits (Dalgleish et al., 2020; Grotzinger, 2021).

Together, these call for an integration of the current literature into an empirically-based theoretical framework that would help clarify the role of empathy across diagnostic lines. We recently proposed a novel way to investigate empathy by examining the imbalance between emotional and cognitive empathy (Shalev and Uzefovsky, 2020; Shalev et al., 2022). We showed that this imbalance, termed empathic disequilibrium, is related to autism diagnosis and traits. In the current study, we aimed to expand the understanding of empathy disequilibrium beyond autism and examine whether it relates to symptoms of different psychopathologies, which are prevalent in the general population. We also looked at the hypothesized role of emotion regulation as a mechanism underlying these associations.

Empathy comprises of cognitive and emotional components (Decety and Jackson, 2004). Emotional empathy (EE) refers to our ability to share others' emotional experiences while maintaining a self-other distinction and cognitive empathy (CE) refers to our ability to recognize and understand others' emotional states. As these are essential for socializing and maintaining interpersonal relationships, alterations in empathy have been reported in the majority of psychological conditions, including (but not limited to) autism (Baron-Cohen and Wheelwright, 2004), schizophrenia (Bonfils et al., 2017), anti-social personality disorder (Baron-Cohen, 2013), major depressive disorder (Schreiter et al., 2013), and obsessive-compulsive disorder (Fontenelle et al., 2009).

As EE and CE have different underlying neurobiological and developmental underpinnings (Shamay-Tsoory et al., 2008; De Waal and Preston, 2017), research typically examined each empathic component independently. As a result, most studies highlighted the differential links between EE and CE and various psychopathologies, finding these for most clinical conditions (Supplementary Table S1). For example, in many studies, individuals diagnosed with autism (Gleichgerrcht et al., 2013; Mazza et al., 2014; Rueda et al., 2015; Mul et al., 2018), schizophrenia (Horan et al., 2016; Vistoli et al., 2017; Atoui et al., 2018; Berger et al., 2019; Van Donkersgoed et al., 2019), and major depressive disorder (Schreiter et al., 2013) typically report intact EE and impaired CE. Elevated EE but intact or lower levels of CE were found in patients with obsessive-compulsive disorders (Fontenelle et al., 2009; Kang et al., 2012). Conversely, lack of empathy in individuals with anti-social personality disorder seems to be related to impaired EE rather than CE (Jones et al., 2010; Van Zonneveld et al., 2017; Winter et al., 2017). However, opposing findings are also abundant (Supplementary Table S1), suggesting that CE and EE are not independent in their relationships with various psychopathologies. As such, some authors suggested that a more holistic view of empathy should be adopted (De Waal and Preston, 2017; Guidi and Traversa, 2021; Håkansson Eklund and Summer Meranius, 2021), and the few studies examining this found CE and EE are interdependent.

Indeed, research support that CE and EE influence, interact, and regulate each other (Gilead et al., 2016; Preckel et al., 2018; Lei et al., 2019). When facing others' emotions, the (emotional) empathic response elicits emotions in the perceiver. Consequently, CE may interact with EE in order to down-regulate (e.g., through identifying and labeling the emotion experienced to reduce the intensity of that emotion) and/or up-regulate these emotions (e.g., through facilitating emotional arousal).

This notion gains further support from neuroimaging studies showing that there is a connectivity between EE- and CE-related regions, and these are co-activated when empathizing with other's pain, especially when additional information is required to deduce about other's feelings (Lamm et al., 2011; Torre and Lieberman, 2018). The mutual regulation between CE and EE suggests that the interplay between them, or a balanced response, may be meaningful for an appropriate empathic reaction. On the other hand, when this balance is interrupted, the response to others' emotions may become inappropriate or disruptive. Therefore, we hypothesize that the intra-personal balance or imbalance between CE and EE may be a crucial mechanism for understanding the role of empathy in psychopathologies.

Following this line of thought, we previously defined the term empathic disequilibrium, relating to the degree of intra-individual imbalance between CE and EE (Shalev and Uzefovsky, 2020; Shalev et al., 2022). The dissociation between CE and EE in autism (Gleichgerrcht et al., 2013; Rueda et al., 2015; Mul et al., 2018), together with the inconsistent findings regarding the association between autism and empathy in the literature (Scambler et al., 2007; Aan Het Rot and Hogenelst, 2014; Mazza et al., 2014), led us to apply this concept in autism. By looking at each component separately, previous research typically conceptualized autistic individuals as having reduced CE and intact EE, focusing on the deficits in empathy in autism (Baron-Cohen, 2013). Yet these findings were not always consistent and did not necessarily coincide with the experience of some individuals reporting “excess of empathy” (Gillespie-Lynch et al., 2017). Reexamining these findings through the perspective of empathic disequilibrium provided a new framework for understanding the links between empathy and autism. Indeed, empathic disequilibrium was predictive of autism diagnosis and autistic traits beyond empathy (Shalev and Uzefovsky, 2020; Shalev et al., 2022).

While empathic disequilibrium strongly characterizes autism, it may not be a unique experience shared only by autistic individuals. Of evidence, although typical individuals tend to show, on average, relatively balanced levels of empathy, empathic disequilibrium is prevalent (and is normally distributed) in the typical population (see histogram in Supplementary Figure S1), where it is associated with autistic traits and alexithymia (Shalev and Uzefovsky, 2020). Therefore, other dimensions of clinical conditions could be related to such experience. Accordingly, the framework of empathic disequilibrium may more broadly be informative for understanding the relationship between various psychopathologies and their manifestations, in which there is a marked social difficulty and empathy.

Importantly, previous findings highlighted the role of empathic disequilibrium where EE is relatively higher than CE (from here forth, EE-dominance) in autism, but CE-dominance may play a role in other conditions. EE-dominance, reflects some individuals' tendency to experience others' emotions more strongly than their ability to understand and recognize these emotions. On the other extreme of the empathic disequilibrium range is CE-dominance, which reflects some individuals' tendency to focus on understanding others' emotions while becoming relatively less emotionally engaged with these emotions. Either extreme could potentially lead to difficulties in social communication and may also differ in their outcomes. Indeed, EE-dominance was related to autism diagnosis and social difficulties, while CE-dominance was related to the non-social domain of autism, such as the drive to analyze systems (Shalev and Uzefovsky, 2020; Shalev et al., 2022). This raises the question of how each of the empathic disequilibrium extremes may be related to psychopathologies.

To understand empathic disequilibrium and its effects on social behavior, we must also delve into the mechanisms that could underlie such an association. Thus far, only a few theoretical frameworks have attempted to explain the links between empathy and psychopathology, and each lacked sufficient empirical support. For example, it was previously proposed that extreme (either high or low) levels of empathy trigger emotion dysregulation (Schipper and Petermann, 2013). In addition, the empathy imbalance hypothesis posited that a combination of high EE and low CE results in over-arousal as one becomes overwhelmed with the feelings of others (Smith, 2006, 2009). These theories could help explain, at least partially, why EE-dominance was associated with autism and autistic traits, where over-arousal is common (Conner et al., 2021). We therefore suggest that CE-dominance, where the cognitive understanding of other's emotions surpasses the emotional experience, might lead to reduced emotionality.

Based on these, we expect an association between empathic disequilibrium and emotional reactivity. Emotional reactivity is a core part of the emotional experience, referring to individual variability in the experience of emotion in terms of intensity, responsiveness to stimuli, and duration of arousal (Davidson, 1998). Problems in all aspects of emotional reactivity prevail in most major psychopathologies and are implicated in their development, maintenance, and prognosis (Davidson, 2003; Gross and Jazaieri, 2014; Sheppes et al., 2015; Sturm et al., 2016; Becerra et al., 2019). We expect that the surfeit of emotions experienced in EE-dominance will be related to conditions characterized by hyper-reactivity, such as autism (Conner et al., 2021), anxiety (Tan et al., 2012), and psychosis (Myin-Germeys and Van Os, 2007). Contrarily, we expect CE-dominance to be related to psychopathologies characterized by hypo-reactivity and lack of emotionality, such as anti-social behavior (Babcock et al., 2005).

In the current study, we empirically tested the proposed theoretical model of the association between empathic disequilibrium and a wide range of symptoms or traits related to clinical conditions, including depression, anxiety, psychosis-related, anti-social behavior, obsessive-compulsive disorder, and autism. In line with previous research showing that symptoms of psychopathology lie on a continuum in the population (Markon et al., 2011), these dimensions were measured as continuous variables and assessed in the general population reflecting normative variations in these dimensions.

We hypothesized that EE-dominance and CE-dominance would be associated with symptoms and traits related to different clinical conditions. First, we expected to replicate previous findings showing an association between EE-dominance and the social domain of autism, as well as an association between CE-dominance and the non-social domain of autism (Shalev and Uzefovsky, 2020). We also expected CE-dominance to be associated with symptoms of anti-social behavior.

Second, we hypothesized that EE-dominance would be associated with symptoms of anxiety, psychotic, and obsessive-compulsive disorders. With regards to depression, because some findings suggest that depression is associated with high EE (Gleichgerrcht et al., 2013; Schreiter et al., 2013; Rueda et al., 2015; Mul et al., 2018), while others find associations with reduced emotional reactivity (Bylsma et al., 2008; Hill et al., 2019), which may suggest an association with CE-dominance, we had no clear hypothesis.

As was mentioned above, our hypothesis is that emotional reactivity plays a role in the association between empathic disequilibrium and clinical traits. Accordingly, we expected traits associated with EE-dominance would be linked to heightened emotional reactivity, while traits associated with CE-dominance would be related to lower emotional reactivity. Finally, we expected that the links between empathic disequilibrium and emotional reactivity would partially explain these associations. Importantly, we used a conservative statistical approach to deal with the more exploratory aspects of the current study, as described below.

## Methods

### Participants

This study was approved by the Ethics Committee of Ben-Gurion University. A power analysis based on 5,000 Monte Carlo simulations revealed that 375 participants should provide sufficient power (1-*β* > 0.8, α = 0.05) to detect effects of small-to-medium sizes. Power analysis was conducted using the ‘simr' v1.0.5 package (Green and Macleod, 2016). This study was not pre-registered. However, measures were specifically collected for the purpose of this study. Accordingly, all measures collected were described in this paper.

To ensure sufficient statistical power is achieved, 425 participants (73.53% females, aged 18–72) were recruited from the general population through advertisements placed on social media during October and November 2020. Participants reported regarding their demographics and filled a battery of online questionnaires assessing their empathy, emotional reactivity, and multiple dimensions of psychopathology. Participants were asked if they were previously diagnosed with any psychiatric condition/s, with most (73.53%) reported not having been diagnosed with any clinical condition. Of those who have been diagnosed, major depressive disorder (40.35%) and attention deficit and hyperactivity disorder (47.22%) were the most common diagnoses. Comorbidity was present in 28.70% of diagnosed individuals. To assure participants were paying attention throughout the study, five attention checks (e.g., “sometimes people do not read all the items. If you read this, please mark 4”) were embedded in the questionnaires. Seventeen participants who did not pass two or more attention checks were excluded from the analyses leaving a total of 408 participants.

Missing data in the sample (an average of 8.92%) was found to be missing completely at random using Little's test (*p* = 0.91) conducted in R “naniar” package v0.6.1 (Tierney and Cook, 2023). Missing data was then completed using a multiple imputations method creating five imputations via the “mice” package v3.13.0 (Van Buuren and Groothuis-Oudshoorn, 2011). All results were pooled using Rubin's rule (Rubin, 2004).

### Measures

#### Empathy

We measured empathy using the Interpersonal Reactivity Index (IRI; Davis, 1980). The questionnaire consists of 28 items on a five-point scale, which can be divided into four validated subscales, each made up of seven items. Two subscales tap CE (“perspective taking” and “fantasizing”), and two subscales tap EE (“empathic concern” and “personal distress”). Cronbach's α was 0.72 for the EE scale and 0.78 for the CE scale.

#### Emotion reactivity

Emotion reactivity was measured using the Emotion Reactivity Scale (ERS; Nock et al., 2008). The ERS consists of 21 items, rated on a 0 to 4 scale, and measures three aspects of emotional reactivity: emotion sensitivity (how easily one gets emotional), arousal intensity, and persistence (the time it takes for the emotional reactivity to pass). Cronbach's α for the total scale was 0.95.

#### Autistic traits

To measure autistic traits, we used the Autism-Spectrum Quotient—Short Version (AQ-28; Hoekstra et al., 2011), an abridged version of the Autism-Spectrum Quotient (Baron-Cohen et al., 2001) consisting of 28 items that assess autistic traits. This measure includes two factors. The social behavior factor is calculated as the sum of the social skills, attention switching, a preference for routines, and imagination subscales. The numbers/patterns factor is a non-social factor tapping fascination with numbers and patterns. In the current study, Cronbach's α for the social behavior and the number/patterns factors were 0.77 and 0.74, respectively.

#### Psychopathic traits

Psychopathic tendencies were assessed using two subscales (“callous affect” and “interpersonal manipulation”) of the Self-Report Psychopathy Scale III (SRP-III; Paulhus et al., 2009). Each subscale contains 16 items rated on a five-point Likert scale, with higher scores indicating a higher psychopathic tendency. Cronbach's α for this measure was 0.84.

#### Symptoms of psychopathology

Symptoms of psychopathology were assessed using the Brief Symptoms Inventory (BSI; Derogatis and Melisaratos, 1983). The BSI is a 53-item self-report scale of symptoms used in clinical and non-clinical populations. Each item is rated on a five-point scale. To alleviate some of the emotional burden of our battery of questionnaires, we intentionally dropped two items concerning suicidal ideation and thoughts, making this subscale less reactive. We specifically chose the BSI as it maps dimensions of psychopathology (Cronbach's α for each dimension is reported in parenthesis), including subscales of depression (0.89), somatization (0.83), obsessive-compulsive symptoms (0.84), interpersonal sensitivity (0.79), anxiety (0.85), hostility (0.79), phobic anxiety (0.7), paranoid ideation (0.81), and psychoticism (0.75). Each subscale of the BSI is converted to T-scores based on a normative sample (Derogatis and Melisaratos, 1983; Gilbar and Ben-Zur, 2002).

### Statistical analysis

#### Sex differences

To examine mean differences in empathic disequilibrium, we used *t*-tests conducted via the “MKmisc” package v1.8 (Kohl, 2021). Empathic disequilibrium was used as a dependent variable in this analysis and was therefore calculated by subtracting the standardized score of CE from the standardized score of EE, as was recommended by Edwards (1995). One-sided tests were used as we expected to replicate previous results where females showed a slight tendency toward EE-dominance, while males showed a tendency toward CE-dominance (Shalev and Uzefovsky, 2020; Shalev et al., 2022).

#### Response surface analysis of empathy

As described in Shalev et al., 2022, we employed polynomial regression with response surfaces analysis (PRRSA; Edwards, 1994; Shanock et al., 2010). PRRSA allows one to visualize and simultaneously estimate the degree to which similarity and dissimilarity between two variables of interest are associated, both linearly and curvilinearly, with an outcome variable, as defined by the polynomial equation:


$$\begin{array}{l} \text{Z}={\text{b}}_{0}+{\text{b}}_{1}\text{CE}+{\text{b}}_{2}\text{EE}+{\text{b}}_{3}\text{C}{\text{E}}^{2}+{\text{b}}_{4}\text{CE}\times \text{EE}+{\text{b}}_{5}\text{E}{\text{E}}^{2}+\text{e} \end{array}$$

**Figure 1:**
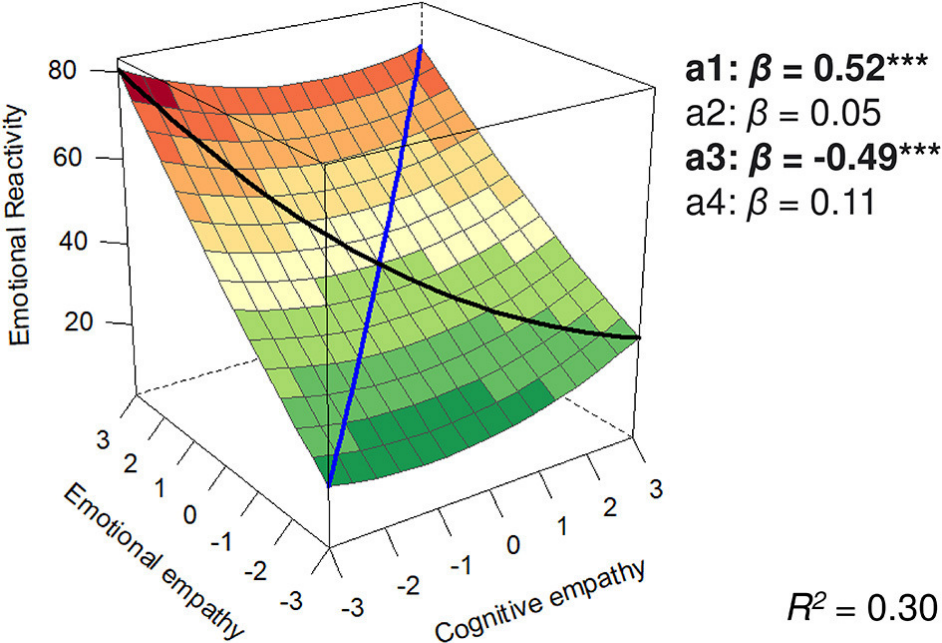
Polynomial regression plot predicting emotional reactivity. The black line represents empathic disequilibrium, and the blue line represents total empathy. ***p < 0.0005.

In line with our hypotheses, parameters of the PRRSA were used to examine both equilibrium (blue line in Figure 1) and disequilibrium (black line in Figure 1) between CE and EE and their relationships with each outcome. These two lines are described by four parameters derived from the equation above estimating the linear (a1) and non-linear (a2) association of overall empathy and the outcome; and the linear (a3) and non-linear (a4) association of empathic disequilibrium with the outcomes. Sex and age were controlled for as covariates. All analyses were also conducted using traditional difference score analyses, which entirely replicated all results (see Supplementary Text).

#### Empathic disequilibrium association with psychopathology symptoms

In this section, we adopted a more exploratory approach. To make sure our findings are robust and replicate we randomly split the dataset into two. The first dataset (30% of the data, *N* = 123) was used to identify the specific dimensions of psychopathology related to empathic disequilibrium. The BSI subscales are highly correlated (see correlation matrix in Supplementary Figure S2), reflecting, in part, general distress levels (Piersma et al., 1994). Therefore, to gain better understanding of the unique dimensions predicted by empathic disequilibrium, each subscale was examined after partialing out all other subscales of the BSI. In the second dataset (70% of the data, *N* = 285), only dimensions that were significantly related to empathic disequilibrium (either linearly or non-linearly) in the first dataset were further examined in the mediation analyses. It should be noted that such approach (partialing-out large proportion of the variance in each BSI scale as well as reducing our sample size), might result in a Type II error (Lavery et al., 2019). However, such a design ensures an independent replication of any association signals detected.

#### Mediation analyses

To test for mediation, we followed the guidelines suggested by Yzerbyt et al. (2018), examining the significance of the association between the PRRSA parameters and emotional reactivity (path A) and the association between emotional reactivity and symptoms/traits controlling for the PRRSA parameters (path B). The product of paths A and B was used to estimate the indirect path (path AB), calculating 95% confidence intervals using Monte-Carlo resampling of 10,000 samples via the MonteCarloCI function of the “semTools” package v0.5-5 (Jorgensen et al., 2016). Sex and age were controlled for as covariates. The social and non-social autistic behaviors were examined within the same analysis to control for a possible covariance between the two scales.

PRRSA parameters assessment and the mediation analyses were conducted using the “lavaan” package v0.6.9 (Rosseel et al., 2017). Response surfaces were plotted using the plotRSA function of the RSA package v0.10.4 (Schönbrodt and Humberg, 2023). All analyses were performed in R v4.1.2 (R Core Team, 2020).

## Results

Descriptive statistics of the sample appear in Table 1 and the zero-order correlations appear in Supplementary Figure S2. The residuals of all regression models examined were inspected and were found to be approximately normally distributed. No multicollinearity was detected as indicated by the Variance Inflation Factor (VIF) values (see Supplementary Figure S3).

**Table 1:** Descriptive statistics of the sample and the measures used.

	**Measure**	**Mean/quantity (SD/frequency)**
Sex		300 females (73.53%)
Age		29.47 (11.17)
Family status	Single	301 (73.95%)
	Married	100 (24.57%)
	Divorced	5 (1.23%)
	Widowed	1 (0.25%)
Education	Less than high school diploma	3 (0.74%)
	High school diploma	198 (48.65%)
	Undergraduate student	161 (39.55%)
	Graduate student	45 (11.06%)
Socioeconomic status	Much less than average	34 (8.37%)
	Less than average	48 (11.82%)
	Average	138 (33.99%)
	More than average	148 (36.45%)
	Much more than average	38 (9.37%)
Diagnoses	No diagnosis	300 (73.53%)
	ADHD	51 (12.5%)
	Major depressive disorder	47 (11.52%)
	Obsessive-compulsive disorder	15 (3.68%)
	Anxiety disorders	12 (2.94%)
	Eating disorders	8 (1.96%)
	Autism	1 (0.25%)
	Personality disorders	1 (0.25%)
IRI	Empathic concern	19.21 (3.86)
	Personal distress	13.52 (4.37)
	Perspective taking	19.11 (4.21)
	Fantasizing	16.42 (5.14)
BSI		50 (10)
ERS		39.23 (18.26)
SRP	Total	70.33 (13.32)
	Callous unemotional	32.82 (7.42)
	Interpersonal manipulation	37.51 (7.78)
AQ	Switching	9.43 (2.58)
	Imagination	16.48 (3.34)
	Social Skill	13.94 (4.06)
	Routine	9.59 (2.36)
	Numbers/patterns	11.63 (3.51)

IRI, Interpersonal Reactivity Index; BSI, Brief Symptom Inventory; ERS, Emotion Reactivity Scale; SRP, Self-Report Psychopathy; AQ, Autism-Spectrum Quotient; ADHD, Attention Deficit and Hyperactivity Disorder.

A *t*-test revealed sex differences in empathic disequilibrium (*t* = 2.26, *p* = 0.01, $\eta_{\text{p}}^{2}$ = 0.01). In line with our previous work (Shalev et al., 2022), empathic disequilibrium in males (mean = 0.19, *SD* = 1.05) differed from balanced empathy (CE – EE = 0) showing a tendency toward CE-dominance (*t* = 1.91, *p* = 0.03, $\eta_{\text{p}}^{2}$ = 0.03), unlike females (mean = −0.07, *SD* = 1.03) who did not differ from balanced empathy (*t* = −1.19, *p* = 0.12, $\eta_{\text{p}}^{2}$ = 0.005).

In the following sections, the PRRSA models and their surface parameters (a1-a4, as described in the Methods Section) are described. For convenience and transparency, raw estimates, their confidence intervals, and full *p*-values of the surface parameters are described within the text as well as all parameters of the mediation analyses (ab path). Standardized estimates (*β*) and proportion of variance explained (*R*^2^) are displayed in the corresponding figures. The parameters of the polynomial regression (b1–b5) for all models are shown in Supplementary Table S2.

To examine whether emotional reactivity may mediate the associations between empathic disequilibrium and psychopathologies, we first examined whether empathic disequilibrium is associated with emotional reactivity (Figure 1). Total empathy and empathic disequilibrium were linearly related to emotional reactivity [*b*_a1_ = 9.39, 95% CI (7.46, 11.31), *p* < 0.0001; *b*_a3_ = −9.01, 95% CI (−11.95, −6.07), *p* < 0.0001], no curvilinear association were found [*b*_a2_ = 0.55, 95% CI (−0.89, 1.99), *p* = 0.46; *b*_a4_ = 1.57, 95% CI (−2.09, 5.23), *p* = 0.40].

### Autistic traits

Overall, and in line with previous findings (Shalev and Uzefovsky, 2020; Shalev et al., 2022), empathic disequilibrium, but not empathy, was associated with both social and the non-social domain of autism.

**Figure 2:**
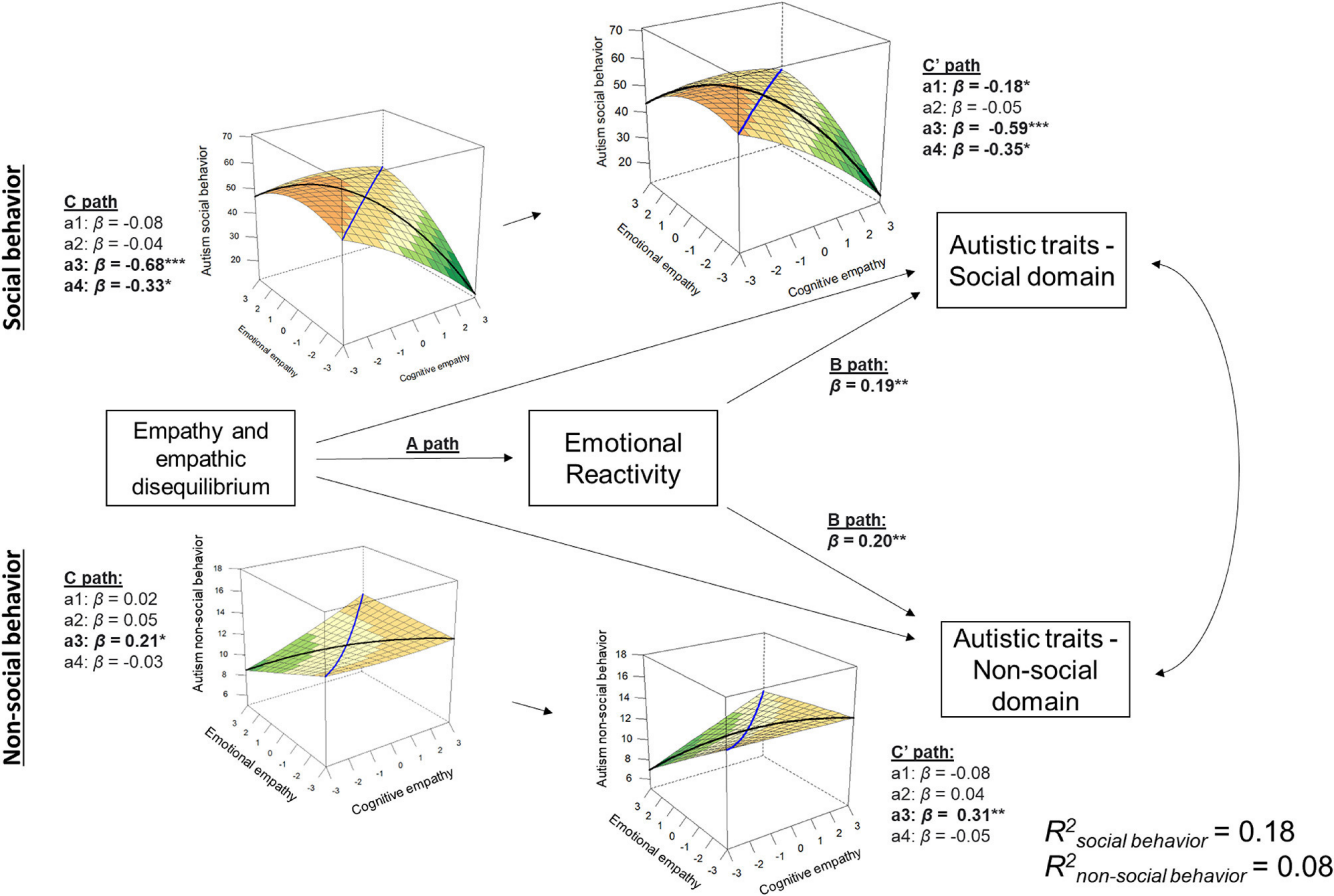
Mediation model for autistic traits. This plot shows the association between the response surface analysis and the social and non-social autistic traits and its mediation by emotional reactivity. Response surface plots for the total effect (C path) and direct effect (C′ path) association are depicted. The black line in the response surface plots represents empathic disequilibrium, and the blue line represents total empathy. *p < 0.05, **p < 0.005, ***p < 0.0005.

A tendency toward EE-dominance was strongly associated with the social domain of autism [*b*_a3_ = −5.57, 95% CI (−7.07, −4.07), *p* < 0.0001], although this decreased at higher levels of empathic disequilibrium as suggested by the non-linear association [*b*_a4_ = −2.15, 95% CI (−4.03, −0.27), *p* = 0.03; Figure 2]. This linear association was partially mediated by emotional reactivity [*β*_ab_ = −0.09, ab path estimate = −0.77, 95% CI (−1.33, −0.30); *b*_a3_ = −4.80, 95% CI (−6.34, −3.26), *p* < 0.0001]. No total effect was found between overall empathy and the social behavior domain of autism [*b*_a1_ = −0.65, 95% CI (−1.63, 0.34), *p* = 0.20]. However, once emotional reactivity was included in the model, a linear association between overall empathy and social domains of autism emerged [*b*_a1_ = −1.45, 95% CI (−2.52, −0.38), *p* = 0.008; *β*_ab_ = 0.10, ab estimate = 0.80, 95% CI (0.33, 1.31)].

Contrarily, in the non-social domain (Figure 2), a linear association with empathic disequilibrium toward CE-dominance was found [*b*_a3_ = 0.73, 95% CI (0.06, 1.41), *p* = 0.03]. This association grew stronger once we entered emotional reactivity into the model [*β*_ab_ = −0.10, ab estimate = −0.34, 95% CI (−0.60, −0.13); *b*_a3_ = 1.07, 95% CI (0.38, 1.78), *p* = 0.003]. No associations (linear or non-linear) were found between total empathy and the non-social domain of autistic traits [*b*_a1_ =0.07, 95% CI (−0.38, 0.51), *p* = 0.78; *b*_a2_ = 0.16, 95% CI (−0.17, 0.49), *p* = 0.35].

### Psychopathic tendencies

**Figure 3:**
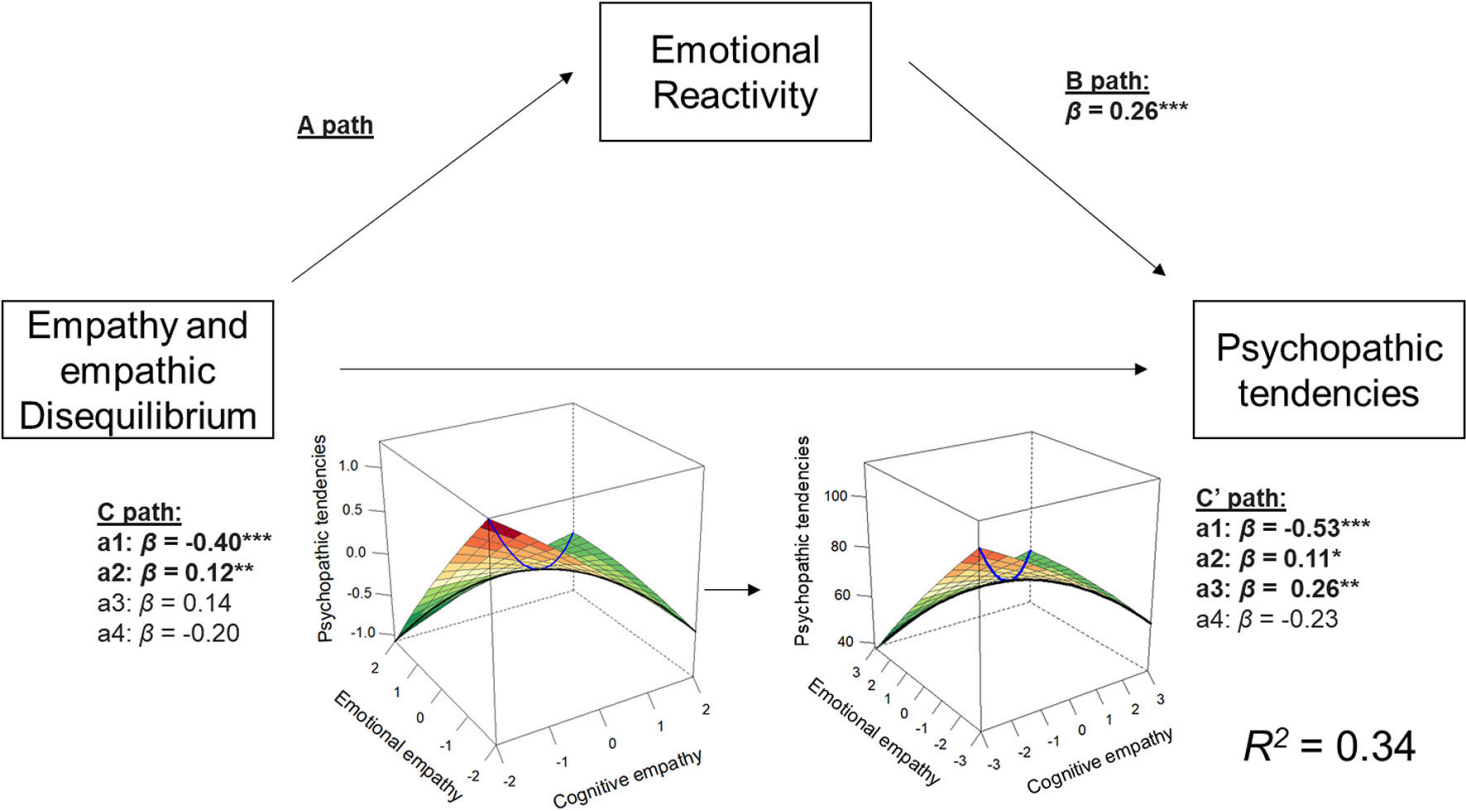
Mediation model for psychopathic tendencies. This plot shows the association between the response surface analysis and psychopathic tendencies and its mediation by emotional reactivity. Response surface plots for the total effect (C path) and direct effect (C′ path) association are depicted. The black line in the response surface plots represents empathic disequilibrium, and the blue line represents total empathy. *p < 0.05, **p < 0.005, ***p < 0.0005.

Although the total effect of the association between empathic disequilibrium and psychopathic tendencies was insignificant [*b*_a3_ = 1.80, 95% CI (−0.35, 3.95), *p* = 0.10], once emotional reactivity was entered into the model, the linear effect between empathic disequilibrium and psychopathic tendencies became significant [*β*_ab_ = −0.13, ab estimate = −1.68, 95% CI (−2.58, −0.92); *b*_a3_ = 3.48, 95% CI (1.31, 5.64), *p* = 0.002]. Thus, controlling for emotional reactivity brings to light the association between psychopathic tendencies and CE-dominance (Figure 3). Overall empathy was linearly related to psychopathic tendencies [*b*_a1_ = −5.32, 95% CI (−6.73, −3.91), *p* < 0.0001], and controlling for emotional reactivity increased this association [*β*_ab_ = 0.13, ab estimate = 1.71, 95% CI (1.05, 2.53); *b*_a1_ = −7.07, 95% CI (−8.57, −5.56), *p* < 0.0001]. Overall empathy also showed a curvilinear association with psychopathic tendencies [*b*_a2_ = 1.50, 95% CI (0.45, 2.56), *p* = 0.005] suggesting higher psychopathic traits are related to the extremes of empathy (i.e., very high or very low empathy).

### Psychopathology symptoms

**Figure 4:**
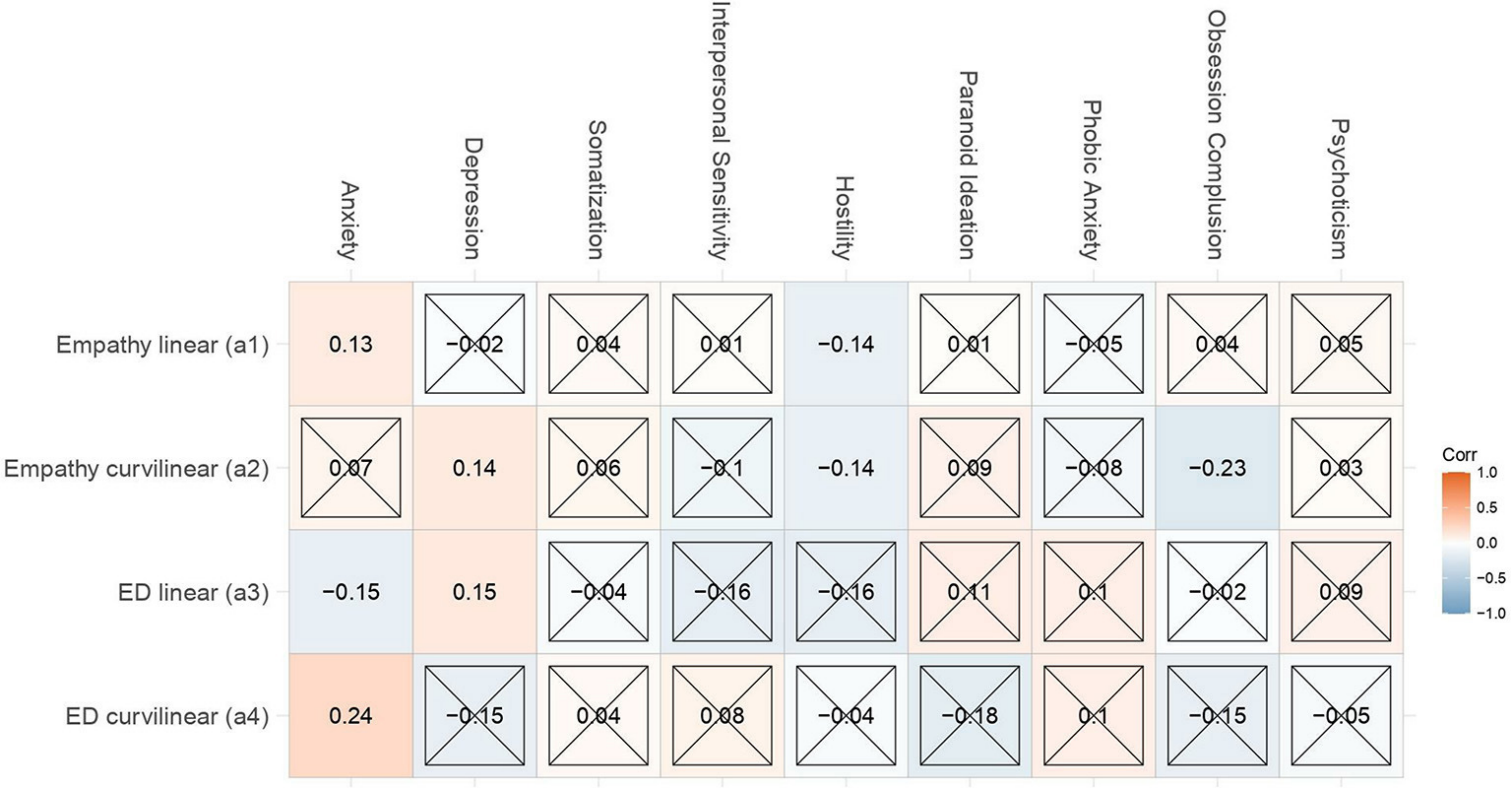
Empathy and empathic disequilibrium association with psychopathology. Standardized correlations between response surface parameters and each psychopathology, controlling for all other dimensions of the Brief Symptom Inventory (BSI) in randomly selected 30% of the data. Insignificant results are concealed by an “X.” All other results have a p-value < 0.05.

We next aimed to examine what dimensions of psychopathology are related to empathic disequilibrium. Thirty percent of the data were randomly selected to examine the partial correlation between the response surface parameters and each specific psychopathology, controlling for all other dimensions of the BSI (see heatmap in Figure 4).

As visible in the heatmap, beyond all other dimensions of psychopathology, empathic disequilibrium with a tendency toward EE-dominance was linearly associated with anxiety [*b*_a3_ = −1.64, 95% CI (−3.06, −0.21), *p* = 0.02], while a tendency toward CE-dominance was related to depression [*b*_a3_ = 1.70, 95% CI (0.28, 3.11), *p* = 0.02]. A non-linear association with anxiety was also significant suggesting that both extremes of empathic disequilibrium are related to anxiety [*b*_a4_ = 2.18, 95% CI (0.49, 3.86), *p* = 0.01].

**Figure 5:**
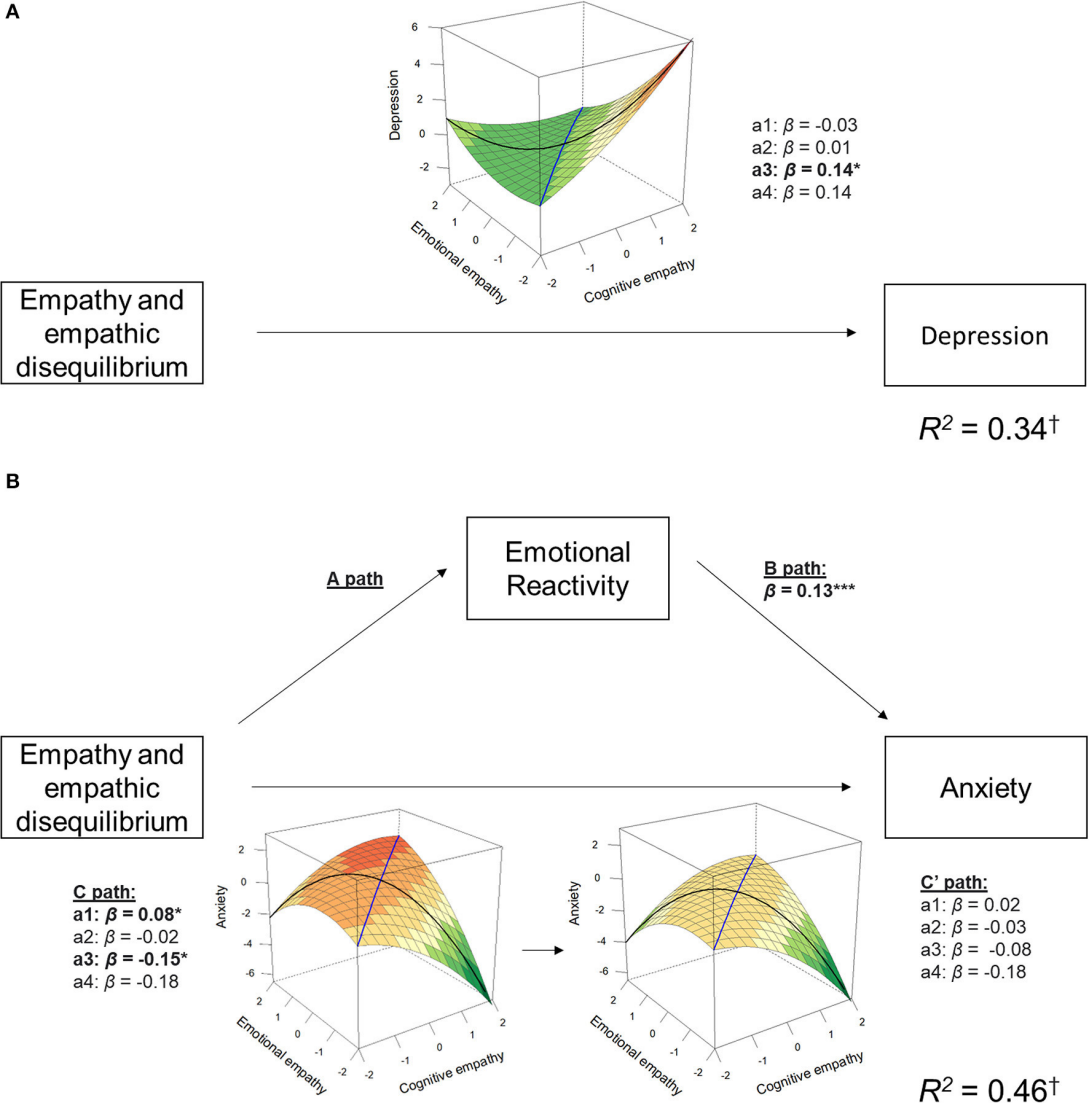
Response surface and mediation models for dimensions of psychopathology. This plot shows the association between the response surface parameters of depression **(A)** and anxiety **(B)**, and the mediation by emotional reactivity for anxiety. Response surface plots for the total effect (C path) and direct effect (C† path) association are depicted. The black line in the response surface plots represents empathic disequilibrium, and the blue line represents total empathy. *p < 0.05, **p < 0.005, ***p < 0.0005. To provide a more representative measure of the variance captured by the response surface and emotional reactivity, *R*^2^ was calculated without controlling for all other BSI dimensions.

Using the remaining 70% of the data we explored whether these associations replicate and whether they are mediated by emotional reactivity. The linear association between empathic disequilibrium and depression was replicated [*b*_a3_ = 1.36, 95% CI (0.27, 2.45), *p* = 0.01]. Yet, such association was not mediated by emotional reactivity as was indicated by an insignificant B path [*b*_B_ = −0.01, 95% CI (−0.05, 0.02), *β*_B_ = −0.02, *p* = 0.46] and as supported by the confidence interval of the indirect path [*β*_ab_ = 0.02, ab estimate = 0.14, 95% CI (−0.24, 0.55)]. That is, depression is directly associated with empathic disequilibrium toward CE-dominance. Visualization of the response surfaces is shown in Figure 5A.

The linear association between empathic disequilibrium (toward EE-dominance) and anxiety was also replicated [*b*_a3_ = −1.34, 95% CI [−2.39, −0.30), *p* = 0.01], as well as the linear association with overall empathy [*b*_a1_ = 0.68, 95% CI (0.04, 1.33), *p* = 0.04]. Both associations were fully mediated by emotional reactivity [*β*_ab_ = −0.08, ab estimate = −1.34, 95% CI (−2.389, −0.30) for empathic disequilibrium; *β*_ab_ = 0.06, ab estimate = 0.56, 95% CI (0.25, 0.90) for overall empathy; see Figure 5B]. The non-linear association with empathic disequilibrium found in the first dataset was not replicated (*b*_a4_ = −1.24, 95% CI (−2.60, −0.14), *p* = 0.08].

## Discussion

This is the first study to examine the intra-individual balance between CE and EE, i.e., empathic disequilibrium, to better understand psychopathology symptoms in the general population. Specifically, we aimed to examine whether empathic disequilibrium can inform our understanding of the role of empathy in various dimensions of clinical conditions and examine a potential mechanism for this association by investigating the role of emotional reactivity as a mediator. First, we found relationships between both extremes of empathic disequilibrium and dimensions of various clinical conditions. Specifically, EE-dominance was related to the social domain of autism and anxiety; and CE-dominance was related to the non-social domain of autism, depression, and psychopathic tendencies. Other clinical-related traits (hostility and obsessive-compulsive symptoms) were related to overall empathy only. We also showed that the associations between empathic disequilibrium and the social domain of autism and anxiety were mediated by emotional reactivity, and that including emotional reactivity in the models increased the direct association between empathic disequilibrium and the non-social domain of autism and psychopathic tendencies. The association with depression was not mediated by emotional reactivity.

Across different dimensions of psychopathology, this study supports the unique contribution of empathic disequilibrium, beyond empathy, to our understanding of clinical conditions. For instance, depressive symptoms and autistic traits were uniquely related to empathic disequilibrium, while hostility and obsessive-compulsive symptoms were only related (non-linearly) to overall empathy. This supports our view that empathic disequilibrium is a distinguishable aspect of empathy that has cross-diagnostic value and could thus provide new means for investigating the relationship between empathy and psychopathology.

Importantly, each extreme of the disequilibrium was predictive of different traits. Replicating previous findings, EE-dominance was related to the social domain of autism, while CE-dominance was related to the non-social domain of autism (Shalev and Uzefovsky, 2020; Shalev et al., 2022). Beyond that, different dimensions of psychopathology were uniquely associated with each type of empathic disequilibrium—EE-dominance was related to anxiety, while CE-dominance was related to depression and psychopathic tendencies.

### Associations with EE-dominance

In addition to the association with the social domain of autism, and in line with our hypotheses, EE-dominance was associated with anxiety. Maladaptive management of emotions has been previously linked to anxiety disorders (Mennin et al., 2007). The association with empathic disequilibrium might suggest that such maladaptive coping with emotions also occurs when perceiving the emotions of others, and that this contributes to anxiety beyond own emotional arousal. This is also in line with neuroimaging studies showing anxiety is related to weaker functional connectivity between CE and EE-related brain regions, such as the connectivity between the ventromedial prefrontal cortex and the amygdala, as well as to altered activation in the anterior insula—a hub region that is commonly activated during CE and EE-related tasks (Shin and Liberzon, 2010; Kim et al., 2011; Marsh, 2018).

### Associations with CE-dominance

In line with our hypotheses, CE-dominance was associated with the non-social domain of autism, psychopathic tendencies, and depressive symptoms. CE-dominance reflects an ability to understand others' feelings accompanied by a relatively muted emotional resonance with these feelings, which could ultimately result in diminished emotionality. Indeed, psychopathic tendencies are related to reduced emotionality (Babcock et al., 2005). CE-dominance was also related to depressive symptoms. The experience of understanding but not feeling for the other may result in feelings of loneliness and emptiness. Therefore, the role of loneliness and emptiness, which often characterize depression (Blatt, 2004; Klonsky, 2008), may mediate this association and this pathway should be examined in future studies.

In contrast to our hypotheses, empathic disequilibrium was not associated with psychotic experiences or with OCD. Our sample did not include a clinical population diagnosed on the psychotic spectrum, and unlike other clinical dimensions such as depression and anxiety, it is debated whether psychotic experiences can be easily measured in the general population (David, 2010). Therefore, it is difficult to conclude whether the null effect reflects a lack of variance in the typical population or a true null. Interestingly, Ciompi (1988, 2014) theorized that schizophrenia is characterized by poor links between emotion and cognition, such as those occurring in empathic disequilibrium. This suggests that it would be important to investigate this association in a clinical sample.

Empathic disequilibrium was also unrelated to symptoms of obsessive-compulsive disorder. Unlike disorders on the psychotic spectrum, studies examining the relationship between empathy and obsessive-compulsive disorder are scarce (Jansen et al., 2020). Yet, the current findings show a non-linear association between empathy and obsessive-compulsive symptoms, which suggests that empathy, but not empathic disequilibrium, is also involved in obsessive-compulsive disorder.

### Emotion reactivity as a mediator or a suppressor

Besides mapping the relationship between empathic disequilibrium and various psychopathologies, we show that emotional reactivity plays a role in this association. The findings suggest two types of mechanisms. Emotional reactivity mediated the association between EE-dominance and anxiety as well as the social domain of autism. This supports our hypothesis and is in line with previous theoretical accounts (Smith, 2006, 2009; Schipper and Petermann, 2013), proposing that such empathic imbalance might cause one to feel overwhelmed by the emotions of others, i.e., experience emotion dysregulation, and that such experience could, in turn, be related to social or emotional difficulties. On the other hand, for the non-social domain of autism and psychopathic tendencies, which were associated with CE-dominance, emotional reactivity did not mediate these associations, but rather controlling for emotion reactivity emphasized or even brought to light the direct associations between these traits and empathic disequilibrium.

Emotional reactivity is intertwined with emotional regulation, a process by which one activates, implicitly or explicitly, a goal to influence their own emotions (Gross, 2015). Different theoretical frameworks suggest that emotional reactivity might be caused by, trigger, or even be indistinguishable from emotional regulation (Nock et al., 2008; Gross and Feldman Barrett, 2011; Gross and Jazaieri, 2014). Emotion regulation and empathy are interrelated as well, and some consider emotion regulation to be one of the components of empathy (Decety, 2010; Thompson et al., 2019). This idea is somewhat reflected in the empirical literature, often treating emotion regulation as a moderator of the relationship between empathy and its possible outcomes, such as psychological distress (Powell, 2018), prosocial behavior (Lockwood et al., 2014), and depressive symptoms (Tully et al., 2016). However, as emotion regulation in these studies is often broadly defined, such interactions cannot specifically inform regarding the interpersonal nature of emotion regulation in empathy. The emotions experienced as part of the empathic response involve a specific form of interpersonal emotion regulation where emotions evoked by others are the subject of the emotion regulatory processes (Zaki, 2020). Thus, we suggest that empathic disequilibrium might serve as a quantitative index for interpersonal emotion dysregulation.

## Limitations

In the current study, we examined mediation models in which empathic disequilibrium predicted emotion regulation and that, in turn, predicted clinically-related traits. As this is a cross-sectional study, we are unable to provide any causal inferences from our data. Our choice of modeling these associations as we did rely on theories linking empathy, clinical conditions, and emotional reactivity as described above (Smith, 2006, 2009; Schipper and Petermann, 2013). Additionally, we used empathy as a predictor because EE and CE are considered basic processes that appear very early in development (De Waal and Preston, 2017; Davidov et al., 2021), while the clinical dimensions examined in our study (except for autism) typically appear later in life and constitute higher-order constructs (Rapee, 2002; Sutin et al., 2013), making our theoretical assumption plausible.

Another limitation of our study is the reliance on self-report measures. While the selected measures are validated and correlate with other behavioral measures (Davis, 1980; Paulhus et al., 2009; Hoekstra et al., 2011; Tew et al., 2015; Derogatis, 2017), they primarily reflect the participants' perception of their own functioning and ability. It should be noted that empathy is largely an internal-experiential process that cannot be readily inferred from behavior alone (Hoffman, 2001), suggesting that self-reports are valuable for understanding empathy. However, future research could benefit from applying behavioral measures to psychopathology and emotional reactivity.

We were also hampered by the high correlations between the psychopathology dimensions measured using the BSI (see Supplementary Figure S2). The BSI measures a wide range of discrete symptoms, shows excellent psychometrics, and is commonly used in both clinical and scientific settings (Derogatis, 2017). However, as it tends to measure the psychological distress associated with each symptom (Piersma et al., 1994), the discrimination between dimensions becomes harder, thus reducing our ability to find strong correlations between empathic disequilibrium and the BSI. Future studies should consider specific measures for each clinical condition. Moreover, examining the same associations in clinical populations and accounting for comorbidity between conditions is also warranted. For example, it should be examined whether the tendency to show empathic disequilibrium in autistic people (Shalev et al., 2022) could be explained (at least partially) by the high rates of anxiety prevalent in the autistic population (Kent and Simonoff, 2017).

Lastly, consistent with earlier studies (Shalev and Uzefovsky, 2020; Shalev et al., 2022), we found that empathic disequilibrium differed between females and males. Despite controlling for sex in all our analyses, we were unable to explore further potential sex differences due to the biased sex ratio in the current sample. Future studies should strive to recruit a more balanced sample of both males and females to accurately assess whether sex moderates the associations between empathic disequilibrium and psychopathology.

## Conclusions

Despite abundant evidence that empathy is related to various clinical conditions, the findings were inconsistent, making it hard to create a unifying theory. In contrast, the concept of empathic disequilibrium so far is very consistent, at least with regard to autism—as this is the third study replicating the association between empathic disequilibrium and autistic traits (Shalev and Uzefovsky, 2020; Shalev et al., 2022). Moreover, it allows to go beyond conceptualizations of the role of empathy in clinical conditions in terms of weaknesses and deficits. Rather, the current findings allow for a more mechanistic understanding by linking the basic mechanisms of empathic disequilibrium and emotional reactivity with specific manifestations in different clinical conditions and as such, also has a transdiagnostic value.

Beyond that, it can directly contribute to the design of clinical interventions. That is, instead of focusing on increasing overall or a specific subcomponent of empathy, clinicians should first map the levels of EE and CE and whether there is an imbalance between them and then tackle that. In the clinical formulation, this can also help clinicians identify clients' specific emotional triggers that bring about emotion dysregulation (e.g., how much one is affected by other's emotional state). Stemming from that are also pathways for ameliorating dysregulation. For example, a person with a relative overabundance of EE may work on increasing self-other distinction and taking care to more accurately understand social situations. On the other hand, a person with a relative overabundance of CE may work on becoming more emotionally attuned to other's emotions. In either case, this will help pinpoint emotional and behavioral triggers and, as such, ameliorate distress.

At a broader level, as empathic disequilibrium represents a different way of experiencing empathy, rather than a reduced or impaired empathy, it could help reduce stigma and improve acceptance for individuals who have previously been perceived as lacking empathy, such as autistic people (Nicolaidis et al., 2018).

Although future research is necessary to determine the directionality of the links between empathic disequilibrium and its outcomes, targeting (or acknowledging) empathic disequilibrium could prove beneficial in dealing with depression, anxiety, or managing overwhelming or underwhelming feelings. Finally, along with emotional reactivity playing a possible role in its underlying mechanism, the conceptualization of empathic disequilibrium brings us closer to a unified theoretical framework that sews together empathy, emotion reactivity, and psychopathology.

## Data availability statement

The datasets presented in this study can be found in online repositories. The names of the repository/repositories and accession number(s) can be found below: https://osf.io/yxcbg/?view_only=dc6408502f8f4054ada7ebe71a714f39.

## Ethics statement

The studies involving human participants were reviewed and approved by Institutional Review Board of Ben-Gurion University. The patients/participants provided their written informed consent to participate in this study.

## Author contributions

Conceptualization and methodology: IS, FU, and AE. Formal analysis, writing—original draft, and visualization: IS. Investigation: IS and FU. Writing—review and editing and supervision: FU and AE. Project administration: FU. All authors contributed to the article and approved the submitted version.
