# Supplementary material for: Empathic disequilibrium as a new framework for understanding individual differences in psychopathology
Source: Front Psychol. 2023 May 19;14:1153447. doi: 10.3389/fpsyg.2023.1153447 (PMC10236526; doi:10.3389/fpsyg.2023.1153447)
Supplement: Supplementary file 1 [file Data_Sheet_1.docx]

**Supplementary Figures**

**Figure S1**

*Histogram with kernel density estimation of empathic disequilibrium*


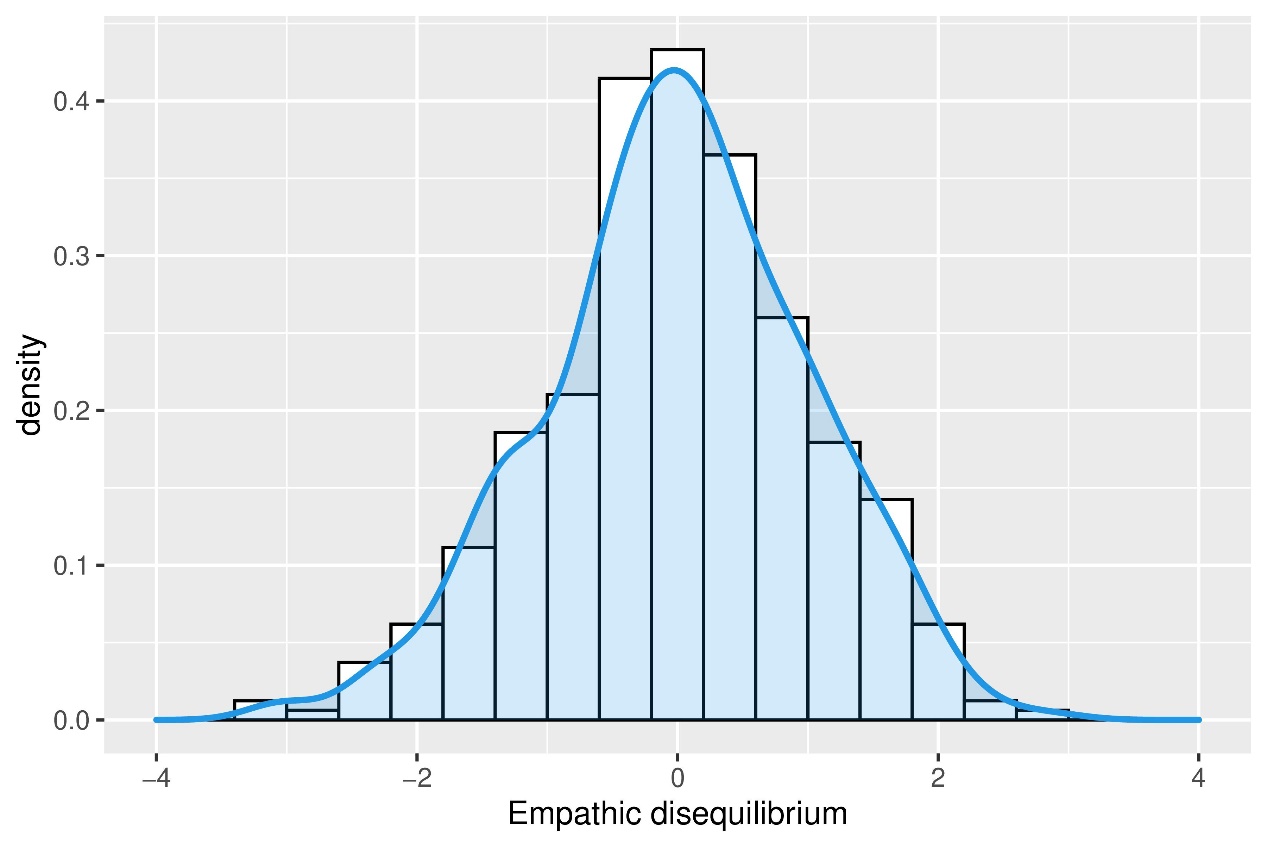


*Notes*. Empathic disequilibrium was calculated as the standardized difference between cognitive and emotional empathy as measured using the Interpersonal Reactivity Index (*N* = 408).

**Figure S2**

*Zero-order Pearson correlations*


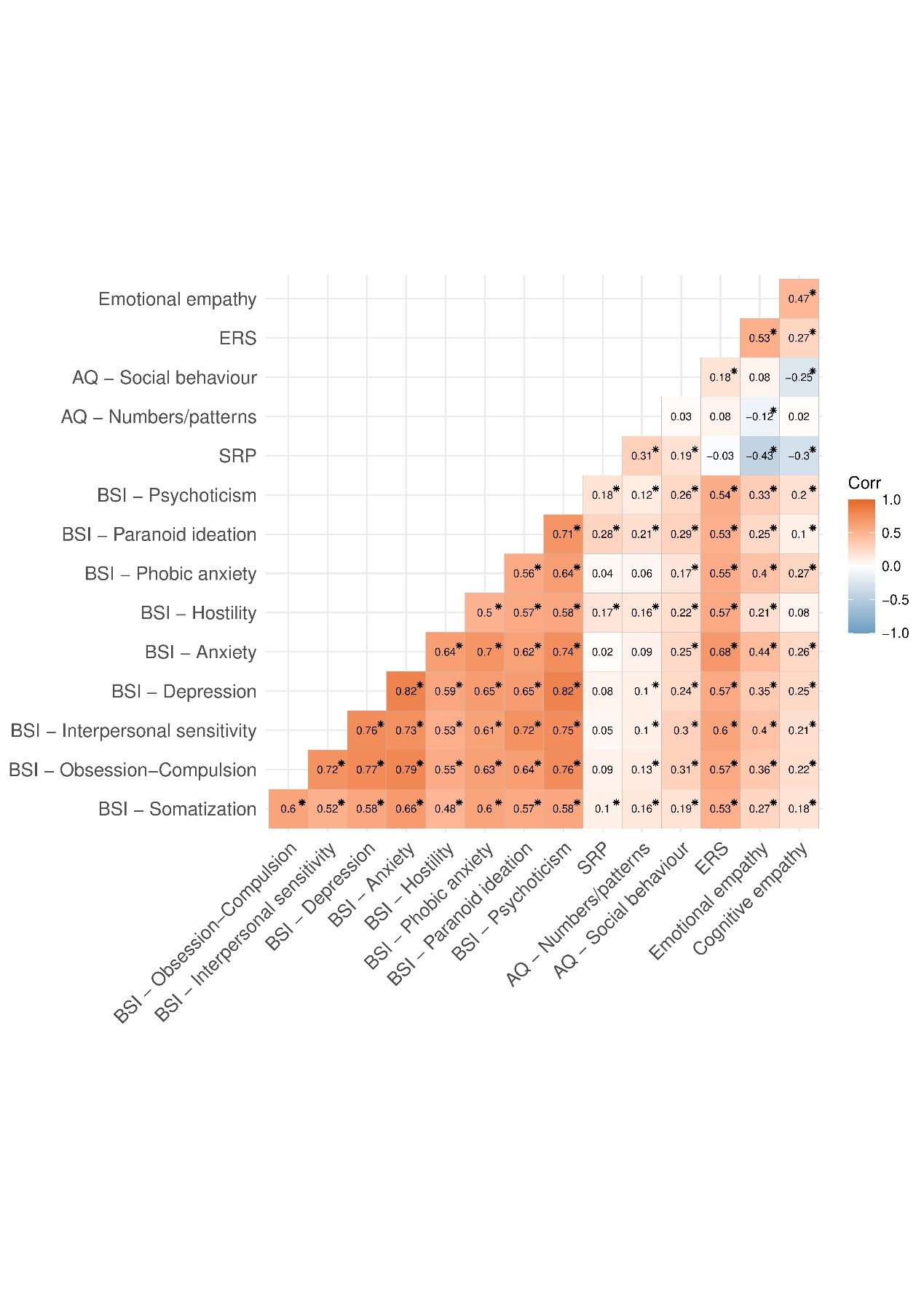


*Notes*. Asterisks indicate significant correlation (*p* < 0.05). AQ – Autism-Spectrum Quotient; SRP – Self-Report Psychopathy; BSI – Brief Symptom Inventory; ERS – Emotion Reactivity Scale. * *p* < 0.05.

**Figure S3**

*Variance Inflation Factor*


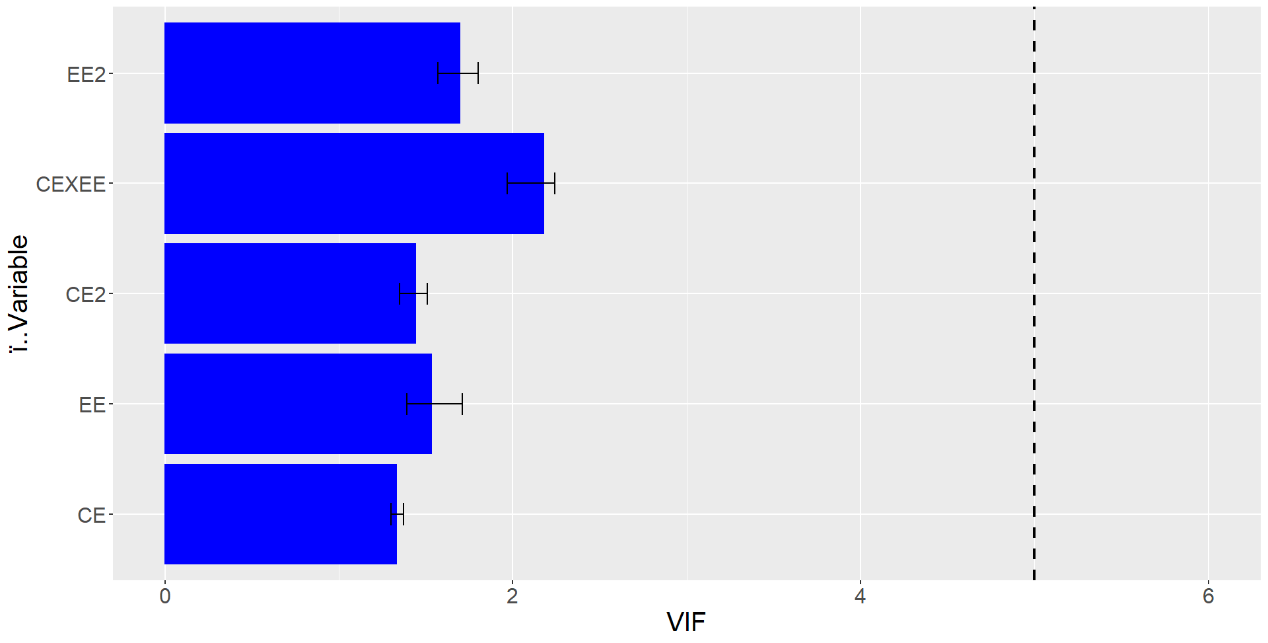

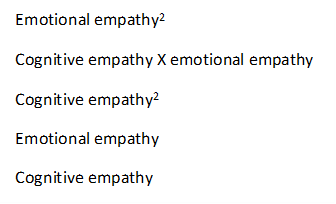

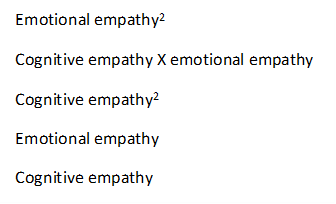

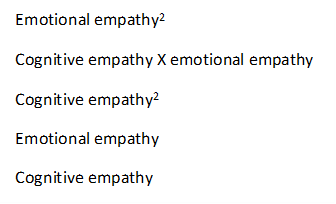

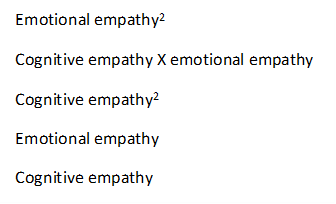

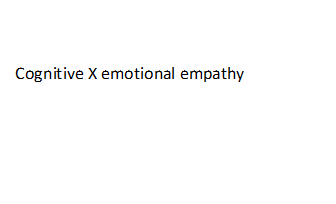


*Notes*. Variance inflation factor (VIF) for each variable of the polynomial regression. Error bars signify the minimum and the maximum values of VIF across all models examined. A value of VIF ≥ 5 (dashed line) indicates serious multicollinearity that requires attention.

**Supplementary Tables**

**Tables S1**

*Examples of typical and mixed findings linking empathy and different psychopathologies*

| Psychopathology | Emotional empathy (e.g., typical findings) | Cognitive empathy (e.g., typical findings) | Null/Opposing evidence |
| --- | --- | --- | --- |
| Autism | **Intact**: Rogers et al. (2007); Dziobek et al. (2008); Gleichgerrcht et al. (2012); Pouw et al. (2013); Deschamps et al. (2014); Rueda et al. (2015); Senland and Higgins-D’Alessandro (2016); Mul et al. (2018); Shirayama et al. (2022) | **Impaired**: Rogers et al. (2007); Dziobek et al. (2008); Oberman et al. (2009); Hirevla and Helkama (2011); Gleichgerrcht et al. (2012); Pouw et al. (2013); Deschamps et al. (2014); Mazza et al. (2014); Adler et al. (2015); Rueda et al. (2015); Senland and Higgins-D’Alessandro (2016); Mul et al. (2018); Bos and Stokes (2019); Shirayama et al. (2022) | **Intact cognitive empathy**: Althaus et al. (2015); Lombardo et al. (2016)  **Impaired emotional empathy:** Scambler et al. (2007); Oberman et al. (2009); Hirevla and Helkama (2011); Adler et al. (2015); Bos and Stokes (2019). |
| Schizophrenia | **Intact:** Achim et al. (2011); Vistoli et al. (2017); Atoui et al. (2018); Berger et al. (2019); van Donkersgoed et al. (2019) | **Impaired:** Benedetti et al. (2009); Abramowitz et al. (2014); Horan et al. (2016); Vistoli et al. (2017); Atoui et al. (2018); Berger et al. (2019); van Donkersgoed et al. (2019) | **Intact cognitive empathy:** Achim et al. (2011); Lehmann et al. (2014); López-del-Hoyo et al. (2019)  **Elevated emotional empathy:** Lehmann et al. (2014); Horan et al. (2016)  **Impaired emotional empathy:** Benedetti et al. (2009); Abramowitz et al. (2014); López-del-Hoyo et al. (2019) |
| Anti-social personality disorder | **Impaired:** Jones et al. (2010); van Zonneveld et al. (2017); Winter et al. (2017) | **Intact:** Richell et al. (2003); Jones et al. (2010); Schiffer et al. (2017); van Zonneveld et al. (2017); Winter et al. (2017) | **Intact emotional empathy:** Shamay-Tsoory et al. (2010); Yavuz et al. (2016); Schiffer et al. (2017)  **Elevated emotional empathy:** Lishner et al. (2012)  **Impaired cognitive empathy:** Shamay-Tsoory et al. (2010); Yavuz et al. (2016) |
| Major depressive disorder | **Intact (empathic concern) or elevated (personal distress):** Thoma et al. (2011); Derntl et al. (2012); Bennik et al. (2019)  **Elevated:** Thoma et al. (2011); Derntl et al. (2012); Bennik et al. (2019) | **Elevated:** Wingenfeld et al. (2016)  **Impaired:** Lee et al. (2005); Cusi et al. (2011); Wolkenstein et al. (2011); Bennik et al. (2019) | **Intact cognitive empathy:** Harkness et al. (2011); Thoma et al. (2011); Wolkenstein et al. (2011); Derntl et al. (2012)  **Impaired emotional empathy:** Cusi et al. (2011); Wingenfeld et al. (2016) |
| Obsessive compulsive disorder | **Elevated:** Fontenelle et al. (2009); Kang et al. (2012); Kim et al. (2018); López-del-Hoyo et al. (2019); Salazar Kämpf et al. (2022) | **Intact:** Fontenelle et al. (2009); López-del-Hoyo et al. (2019); Salazar Kämpf et al. (2022)  **Impaired:** Kang et al. (2012); Kim et al. (2018); López-del-Hoyo et al. (2019) |  |
| Social anxiety disorder | **Elevated**: Tibi-Elhanany and Shamay-Tsoory (2011); Gambin and Sharp (2018) | **Intact:** Campbell et al. (2009); Gambin and Sharp (2018)  **Impaired:** Montagne et al. (2006); Gambin and Sharp (2018); Pepper et al. (2019) | **Elevated cognitive empathy:** Tibi-Elhanany and Shamay-Tsoory (2011)  **Impaired emotional empathy:** Morrison et al. (2016)  **Intact emotional empathy:** Pepper et al. (2019) |

**Table S2**

*Surface parameters for all models*

|  |  | b1  cognitive empathy | b2  emotional empathy | b3  cognitive empathy^2^ | b4 cognitive x emotional empathy | b5  emotional empathy^2^ |
| --- | --- | --- | --- | --- | --- | --- |
| ERS | **A path** | 0.19  [-1.54, 1.92] | **9.2*****  **[7.41, 10.99]** | 0.76  [-0.52, 2.04] | -0.51  [-2.45, 1.43] | 0.30  [-1.11, 1.71] |
| SRP | **C path** | **-1.76***  **[-3.02, -0.50]** | **-3.56*****  **[-4.86, -2.25]** | -0.20  [-1.13, 0.74] | **1.89***  **[0.48, 3.31]** | -0.19  [0.83, -0.19] |
|  | **C' path** | **-1.80****  **[-3.01, -0.58]** | **-5.27*****  **[-6.68, -3.86]** | -0.34  [-1.24, 0.57] | **1.99****  **[0.62, 3.36]** | -0.25  [-1.24, 0.74] |
| AQ - Social Behaviour | **C path** | **-3.11*****  **[-4.00, -2.22]** | **2.46*****  **[1.53, 3.39]** | -0.38  [-1.04, 0.28] | **1.04* [0.03, 2.06]** | -0.72  [-1.45, 0.004] |
|  | **C' path** | **-3.12*****  **[-3.99, -2.26]** | **1.68****  **[0.67, 2.69]** | -0.45  [-1.09, 0.20] | **1.09***  **[0.09, 2.08]** | **-0.75***  **[-1.45, -0.04]** |
| AQ - Numbers/patterns | **C path** | 0.40  [-0.004, 0.80] | -0.33  [-0.75, 0.08] | 0.03  [-0.27, 0.33] | 0.13  [-0.33, 0.59] | 0.002  [-0.33, 0.33] |
|  | **C' path** | **0.40***  **[0.0001, 0.78]** | **-0.68****  **[-1.14, -0.23]** | -0.002  [-0.30, 0.29] | 0.15  [-0.29, 0.59] | -0.01  [-0.33, 0.31] |
| BSI - Depression | **C path** | **0.63***  **[0.005, 1.26]** | **-0.67***  **[-1.29, -0.05]** | 0.17  [-0.32, 0.66] | -0.50  [-1.24, 0.25] | 0.23  [-0.26, 0.73] |
| BSI - Anxiety | **C path** | -0.09  [-0.59, 0.42] | **0.87****  **[0.35, 1.38]** | -0.05  [-0.42, 0.32] | 0.12  [-0.45, 0.68] | 0.06  [-0.35, 0.47] |
|  | **C' path** | -0.36  [-0.97, 0.25] | **0.80***  **[0.18, 1.42]** | -0.31  [-0.79, -0.17] | 0.61  [-0.11, 1.33] | -0.36  [-0.83, 0.12] |

*Notes*. Parameters (b1-b5) of the polynomial regression analyses for all the mediation models. A path represents the association between components of empathy and emotional reactivity (the mediator), the C path represents the total effect (without controlling for emotional reactivity), and the C’ path represents the direct effect after controlling for emotional reactivity. ERS – Emotion Reactivity Scale; SRP – Self-Report Psychopathy; AQ – Autism-Spectrum Quotient; BSI – Brief Symptom Inventory.

* *p* < 0.05, ** *p* < 0.005, *** *p* < 0.0005.

**Supplementary Information References**

Abramowitz, A.C., Ginger, E.J., Gollan, J.K., and Smith, M.J. (2014). Empathy, depressive symptoms, and social functioning among individuals with schizophrenia. *Psychiatry Research* 216**,** 325-332. doi: 10.1016/j.psychres.2014.02.028

Achim, A.M., Ouellet, R., Roy, M.-A., and Jackson, P.L. (2011). Assessment of empathy in first-episode psychosis and meta-analytic comparison with previous studies in schizophrenia. *Psychiatry Research* 190**,** 3-8. doi: 10.1016/j.psychres.2010.10.030

Adler, N., Dvash, J., and Shamay-Tsoory, S.G. (2015). Empathic Embarrassment Accuracy in Autism Spectrum Disorder. *Autism Research* 8**,** 241-249. doi: 10.1002/aur.1439

Althaus, M., Groen, Y., Wijers, A.A., Noltes, H., Tucha, O., and Hoekstra, P.J. (2015). Oxytocin enhances orienting to social information in a selective group of high-functioning male adults with autism spectrum disorder. *Neuropsychologia* 79**,** 53-69. doi: 10.1016/j.neuropsychologia.2015.10.025

Atoui, M., El Jamil, F., El Khoury, J., Doumit, M., Syriani, N., Khani, M., and Nahas, Z. (2018). The relationship between clinical insight and cognitive and affective empathy in schizophrenia. *Schizophrenia Research: Cognition* 12**,** 56-65. doi: 10.1016/j.scog.2018.02.004

Benedetti, F., Bernasconi, A., Bosia, M., Cavallaro, R., Dallaspezia, S., Falini, A., Poletti, S., Radaelli, D., Riccaboni, R., Scotti, G., and Smeraldi, E. (2009). Functional and structural brain correlates of theory of mind and empathy deficits in schizophrenia. *Schizophrenia Research* 114**,** 154-160. doi: 10.1016/j.schres.2009.06.021

Bennik, E.C., Jeronimus, B.F., and Aan Het Rot, M. (2019). The relation between empathy and depressive symptoms in a Dutch population sample. *Journal of Affective Disorders* 242**,** 48-51. doi: 10.1016/j.jad.2018.08.008

Berger, P., Bitsch, F., Jakobi, B., Nagels, A., Straube, B., and Falkenberg, I. (2019). Cognitive and emotional empathy in patients with schizophrenia spectrum disorders: A replication and extension study. *Psychiatry Research* 276**,** 56-59. doi: 10.1016/j.psychres.2019.04.015

Bos, J., and Stokes, M.A. (2019). Cognitive empathy moderates the relationship between affective empathy and wellbeing in adolescents with autism spectrum disorder. *European Journal of Developmental Psychology* 16**,** 433-446. doi: 10.1080/17405629.2018.1444987

Campbell, D.W., Sareen, J., Stein, M.B., Kravetsky, L.B., Paulus, M.P., Hassard, S.T., and Reiss, J.P. (2009). Happy but not so approachable: the social judgments of individuals with generalized social phobia. *Depression and Anxiety* 26**,** 419-424. doi: 10.1002/da.20474

Cusi, A.M., Macqueen, G.M., Spreng, R.N., and Mckinnon, M.C. (2011). Altered empathic responding in major depressive disorder: Relation to symptom severity, illness burden, and psychosocial outcome. *Psychiatry Research* 188**,** 231-236. doi: 10.1016/j.psychres.2011.04.013

Derntl, B., Seidel, E.-M., Schneider, F., and Habel, U. (2012). How specific are emotional deficits? A comparison of empathic abilities in schizophrenia, bipolar and depressed patients. *Schizophrenia Research* 142**,** 58-64. doi: 10.1016/j.schres.2012.09.020

Deschamps, P.K., Been, M., and Matthys, W. (2014). Empathy and empathy induced prosocial behavior in 6- and 7-year-olds with autism spectrum disorder. *J. Autism Dev. Disord.* 44**,** 1749-1758. doi: 10.1007/s10803-014-2048-3

Dziobek, I., Rogers, K., Fleck, S., Bahnemann, M., Heekeren, H.R., Wolf, O.T., and Convit, A. (2008). Dissociation of cognitive and emotional empathy in adults with Asperger syndrome using the Multifaceted Empathy Test (MET). *J. Autism Dev. Disord.* 38**,** 464-473. doi: 10.1007/s10803-007-0486-x

Fontenelle, L.F., Soares, I.D., Miele, F., Borges, M.C., Prazeres, A.M., Rangé, B.P., and Moll, J. (2009). Empathy and symptoms dimensions of patients with obsessive–compulsive disorder. *Journal of Psychiatric Research* 43**,** 455-463. doi: 10.1016/j.jpsychires.2008.05.007

Gambin, M., and Sharp, C. (2018). Relations between empathy and anxiety dimensions in inpatient adolescents. *Anxiety, Stress, & Coping* 31**,** 447-458. doi: 10.1080/10615806.2018.1475868

Gleichgerrcht, E., Torralva, T., Rattazzi, A., Marenco, V., Roca, M., and Manes, F. (2012). Selective impairment of cognitive empathy for moral judgment in adults with high functioning autism. *Social Cognitive and Affective Neuroscience* 8**,** 780-788. doi: 10.1093/scan/nss067

Harkness, K.L., Washburn, D., Theriault, J.E., Lee, L., and Sabbagh, M.A. (2011). Maternal history of depression is associated with enhanced theory of mind in depressed and nondepressed adult women. *Psychiatry Research* 189**,** 91-96. doi: 10.1016/j.psychres.2011.06.007

Hirevla, S., and Helkama, K. (2011). Empathy, values, morality and Asperger’s syndrome. *Scandinavian Journal of Psychology* 52**,** 560-572. doi: 10.1111/j.1467-9450.2011.00913.x

Horan, W.P., Jimenez, A.M., Lee, J., Wynn, J.K., Eisenberger, N.I., and Green, M.F. (2016). Pain empathy in schizophrenia: an fMRI study. *Social Cognitive and Affective Neuroscience* 11**,** 783-792. doi: 10.1093/scan/nsw002

Jones, A.P., Happé, F.G.E., Gilbert, F., Burnett, S., and Viding, E. (2010). Feeling, caring, knowing: different types of empathy deficit in boys with psychopathic tendencies and autism spectrum disorder. *Journal of Child Psychology and Psychiatry* 51**,** 1188-1197. doi: 10.1111/j.1469-7610.2010.02280.x

Kang, J.I., Namkoong, K., Yoo, S.W., Jhung, K., and Kim, S.J. (2012). Abnormalities of emotional awareness and perception in patients with obsessive–compulsive disorder. *Journal of Affective Disorders* 141**,** 286-293. doi: 10.1016/j.jad.2012.04.001

Kim, H.W., Kang, J.I., Hwang, E.H., and Kim, S.J. (2018). Association between glutamate transporter gene polymorphisms and obsessive-compulsive disorder/trait empathy in a Korean population. *PLOS ONE* 13**,** e0190593. doi: 10.1371/journal.pone.0190593

Lee, L., Harkness, K.L., Sabbagh, M.A., and Jacobson, J.A. (2005). Mental state decoding abilities in clinical depression. *Journal of Affective Disorders* 86**,** 247-258. doi: 10.1016/j.jad.2005.02.007

Lehmann, A., Bahçesular, K., Brockmann, E.-M., Biederbick, S.-E., Dziobek, I., Gallinat, J., and Montag, C. (2014). Subjective experience of emotions and emotional empathy in paranoid schizophrenia. *Psychiatry Research* 220**,** 825-833. doi: 10.1016/j.psychres.2014.09.009

Lishner, D.A., Vitacco, M.J., Hong, P.Y., Mosley, J., Miska, K., and Stocks, E.L. (2012). Evaluating the Relation Between Psychopathy and Affective Empathy:Two Preliminary Studies. *International Journal of Offender Therapy and Comparative Criminology* 56**,** 1161-1181. doi: 10.1177/0306624x11421891

Lombardo, M.V., Lai, M.-C., Auyeung, B., Holt, R.J., Allison, C., Smith, P., Chakrabarti, B., Ruigrok, A.N.V., Suckling, J., Bullmore, E.T., Bailey, A.J., Baron-Cohen, S., Bolton, P.F., Bullmore, E.T., Carrington, S., Catani, M., Chakrabarti, B., Craig, M.C., Daly, E.M., Deoni, S.C.L., Ecker, C., Happé, F., Henty, J., Jezzard, P., Johnston, P., Jones, D.K., Lai, M.-C., Lombardo, M.V., Madden, A., Mullins, D., Murphy, C.M., Murphy, D.G.M., Pasco, G., Ruigrok, A.N.V., Sadek, S.A., Spain, D., Stewart, R., Suckling, J., Wheelwright, S.J., Williams, S.C., Ellie Wilson, C., Ecker, C., Craig, M.C., Murphy, D.G.M., Happé, F., Baron-Cohen, S., and Consortium, M.A. (2016). Unsupervised data-driven stratification of mentalizing heterogeneity in autism. *Scientific Reports* 6**,** 35333. doi: 10.1038/srep35333

López-Del-Hoyo, Y., Panzano, M.G., Lahera, G., Herrera-Mercadal, P., Navarro-Gil, M., Campos, D., Borao, L., Morillo, H., and García-Campayo, J. (2019). Differences between individuals with schizophrenia or obsessive-compulsive disorder and healthy controls in social cognition and mindfulness skills: A controlled study. *PLOS ONE* 14**,** e0225608. doi: 10.1371/journal.pone.0225608

Mazza, M., Pino, M.C., Mariano, M., Tempesta, D., Ferrara, M., De Berardis, D., Masedu, F., and Valenti, M. (2014). Affective and cognitive empathy in adolescents with autism spectrum disorder. *Frontiers in Human Neuroscience* 8. doi: 10.3389/fnhum.2014.00791

Montagne, B., Schutters, S., Westenberg, H.G.M., Van Honk, J., Kessels, R.P.C., and De Haan, E.H.F. (2006). Reduced sensitivity in the recognition of anger and disgust in social anxiety disorder. *Cognitive Neuropsychiatry* 11**,** 389-401. doi: 10.1080/13546800444000254

Morrison, A.S., Mateen, M.A., Brozovich, F.A., Zaki, J., Goldin, P.R., Heimberg, R.G., and Gross, J.J. (2016). Empathy for positive and negative emotions in social anxiety disorder. *Behaviour Research and Therapy* 87**,** 232-242. doi: 10.1016/j.brat.2016.10.005

Mul, C.-L., Stagg, S.D., Herbelin, B., and Aspell, J.E. (2018). The Feeling of Me Feeling for You: Interoception, Alexithymia and Empathy in Autism. *Journal of Autism and Developmental Disorders* 48**,** 2953-2967. doi: 10.1007/s10803-018-3564-3

Oberman, L.M., Winkielman, P., and Ramachandran, V.S. (2009). Slow echo: facial EMG evidence for the delay of spontaneous, but not voluntary, emotional mimicry in children with autism spectrum disorders. *Developmental Science* 12**,** 510-520. doi: 10.1111/j.1467-7687.2008.00796.x

Pepper, K.L., Demetriou, E.A., Park, S.H., Boulton, K.A., Hickie, I.B., Thomas, E.E., and Guastella, A.J. (2019). Self-reported empathy in adults with autism, early psychosis, and social anxiety disorder. *Psychiatry Research* 281**,** 112604. doi: 10.1016/j.psychres.2019.112604

Pouw, L.B.C., Rieffe, C., Oosterveld, P., Huskens, B., and Stockmann, L. (2013). Reactive/proactive aggression and affective/cognitive empathy in children with ASD. *Research in Developmental Disabilities* 34**,** 1256-1266. doi: 10.1016/j.ridd.2012.12.022

Richell, R.A., Mitchell, D.G.V., Newman, C., Leonard, A., Baron-Cohen, S., and Blair, R.J.R. (2003). Theory of mind and psychopathy: can psychopathic individuals read the ‘language of the eyes’? *Neuropsychologia* 41**,** 523-526. doi: 10.1016/S0028-3932(02)00175-6

Rogers, K., Dziobek, I., Hassenstab, J., Wolf, O.T., and Convit, A. (2007). Who Cares? Revisiting Empathy in Asperger Syndrome. *Journal of Autism and Developmental Disorders* 37**,** 709-715. doi: 10.1007/s10803-006-0197-8

Rueda, P., Fernández-Berrocal, P., and Baron-Cohen, S. (2015). Dissociation between cognitive and affective empathy in youth with Asperger Syndrome. *European Journal of Developmental Psychology* 12**,** 85-98. doi: 10.1080/17405629.2014.950221

Salazar Kämpf, M., Kanske, P., Kleiman, A., Haberkamp, A., Glombiewski, J., and Exner, C. (2022). Empathy, compassion, and theory of mind in obsessive-compulsive disorder. *Psychology and Psychotherapy: Theory, Research and Practice* 95**,** 1-17. doi: 10.1111/papt.12358

Scambler, D.J., Hepburn, S., Rutherford, M.D., Wehner, E.A., and Rogers, S.J. (2007). Emotional Responsivity in Children with Autism, Children with Other Developmental Disabilities, and Children with Typical Development. *Journal of Autism and Developmental Disorders* 37**,** 553-563. doi: 10.1007/s10803-006-0186-y

Schiffer, B., Pawliczek, C., Müller, B.W., Wiltfang, J., Brüne, M., Forsting, M., Gizewski, E.R., Leygraf, N., and Hodgins, S. (2017). Neural Mechanisms Underlying Affective Theory of Mind in Violent Antisocial Personality Disorder and/or Schizophrenia. *Schizophrenia Bulletin* 43**,** 1229-1239. doi: 10.1093/schbul/sbx012

Senland, A.K., and Higgins-D’alessandro, A. (2016). Sociomoral Reasoning, Empathy, and Meeting Developmental Tasks During the Transition to Adulthood in Autism Spectrum Disorder. *Journal of Autism and Developmental Disorders* 46**,** 3090-3105. doi: 10.1007/s10803-016-2849-7

Shamay-Tsoory, S.G., Harari, H., Aharon-Peretz, J., and Levkovitz, Y. (2010). The role of the orbitofrontal cortex in affective theory of mind deficits in criminal offenders with psychopathic tendencies. *Cortex* 46**,** 668-677. doi: 10.1016/j.cortex.2009.04.008

Shirayama, Y., Matsumoto, K., Hamatani, S., Muneoka, K., Okada, A., and Sato, K. (2022). Associations among autistic traits, cognitive and affective empathy, and personality traits in adults with autism spectrum disorder and no intellectual disability. *Scientific Reports* 12**,** 3125. doi: 10.1038/s41598-022-07101-x

Thoma, P., Zalewski, I., Von Reventlow, H.G., Norra, C., Juckel, G., and Daum, I. (2011). Cognitive and affective empathy in depression linked to executive control. *Psychiatry Research* 189**,** 373-378. doi: 10.1016/j.psychres.2011.07.030

Tibi-Elhanany, Y.M.A., and Shamay-Tsoory, S.G.P. (2011). Social Cognition in Social Anxiety: First Evidence for Increased Empathic Abilities. *The Israel Journal of Psychiatry and Related Sciences* 48**,** 98-106.

Van Donkersgoed, R.J.M., De Jong, S., Aan Het Rot, M., Wunderink, L., Lysaker, P.H., Hasson-Ohayon, I., Aleman, A., and Pijnenborg, G.H.M. (2019). Measuring empathy in schizophrenia: The Empathic Accuracy Task and its correlation with other empathy measures. *Schizophrenia Research* 208**,** 153-159. doi: 10.1016/j.schres.2019.03.024

Van Zonneveld, L., Platje, E., De Sonneville, L., Van Goozen, S., and Swaab, H. (2017). Affective empathy, cognitive empathy and social attention in children at high risk of criminal behaviour. *Journal of Child Psychology and Psychiatry* 58**,** 913-921. doi: 10.1111/jcpp.12724

Vistoli, D., Lavoie, M.-A., Sutliff, S., Jackson, P.L., and Achim, A.M. (2017). Functional MRI examination of empathy for pain in people with schizophrenia reveals abnormal activation related to cognitive perspective-taking but typical activation linked to affective sharing. *Journal of Psychiatry & Neuroscience* 42**,** 262-272. doi: 10.1503/jpn.160136

Wingenfeld, K., Kuehl, L.K., Dziobek, I., Roepke, S., Otte, C., and Hinkelmann, K. (2016). Effects of mineralocorticoid receptor blockade on empathy in patients with major depressive disorder. *Cognitive, Affective, & Behavioral Neuroscience* 16**,** 902-910. doi: 10.3758/s13415-016-0441-4

Winter, K., Spengler, S., Bermpohl, F., Singer, T., and Kanske, P. (2017). Social cognition in aggressive offenders: Impaired empathy, but intact theory of mind. *Scientific Reports* 7**,** 670. doi: 10.1038/s41598-017-00745-0

Wolkenstein, L., Schönenberg, M., Schirm, E., and Hautzinger, M. (2011). I can see what you feel, but I can't deal with it: Impaired theory of mind in depression. *Journal of Affective Disorders* 132**,** 104-111. doi: 10.1016/j.jad.2011.02.010

Yavuz, K.F., Şahin, O., Ulusoy, S., İpek, O.U., and Kurt, E. (2016). Experiential avoidance, empathy, and anger-related attitudes in antisocial personality disorder. *Turkish journal of medical sciences* 46**,** 1792-1800. doi: 10.3906/sag-1601-80
