# Supplementary material for: Empathic disequilibrium as a new framework for understanding individual differences in psychopathology
Source: Front Psychol. 2023 May 19;14:1153447. doi: 10.3389/fpsyg.2023.1153447 (PMC10236526; doi:10.3389/fpsyg.2023.1153447)
Supplement: Supplementary file 2 [file Data_Sheet_2.docx]

**Supplementary text**

**Difference score analyses**

While difference score analysis holds several limitations, it does provide additional information, such as an assessment of the unique contribution of each variable beyond the other, and can help us estimate how much empathic disequilibrium contributes to the prediction of variables beyond total empathy. Therefore, the traditional difference score was reported. Emotional reactivity was associated with both total empathy (*β* = 0.44, *b* = 4.72, 95% CI [3.76, 5.69], *p* < 0.00001) and empathic disequilibrium (*β* = -0.25, *b* = -4.49, 95% CI [-5.95, -3.02], *p* < 0.00001).

**Autistic traits**

The social autistic traits were associated with empathic disequilibrium (*β* = -0.35, *b* = -2.75, 95% CI [-3.49, -2.01], *p* < 0.00001), but not with total empathy (*β* = -0.07, *b* = -0.34, 95% CI [-0.83, 0.15], *p* = 0.15). The association with empathic disequilibrium was partially mediated by emotional reactivity (*β*_ab_ = -0.05, ab estimate = -0.37, 95% CI [-0.64, -0.14]), reducing the strength of the association (*β* = -0.30, *b* = -2.34, 95% CI [-3.14, -1.61], *p* < 0.00001). Emotional reactivity also suppressed the association between empathic disequilibrium and total empathy (*β*_ab_ = 0.08, ab estimate = 0.39, 95% CI [0.16, 0.65]), and an association with total empathy appeared after controlling for emotional reactivity (*β* = -0.15, *b* = -0.73, 95% CI [-1.27, -0.20], *p* = 0.007).

Examination of the non-social domain of autism revealed that it was associated with empathic disequilibrium towards CE-dominance (*β* = 0.11, *b* = 0.37, 95% CI [0.04, 0.71], *p* = 0.03), but not with total empathy (*β* = 0.02, *b* = 0.03, 95% CI [-0.19, 0.25], *p* = 0.77). Emotional reactivity suppressed the association between empathic disequilibrium and autistic traits (*β*_ab_ = -0.05, ab estimate = -0.17, 95% CI [-0.30, -0.07]), increasing its strength (*β* = 0.16, *b* = 0.54, 95% CI [0.20, 0.89], *p* = 0.002). After controlling for emotional reactivity, the association with total empathy remained insignificant (*β* = -0.07, *b* = -0.15, 95% CI [-0.39, 0.09], *p* = 0.23).

**Psychopathic tendencies**

Empathic disequilibrium was not related to psychopathic tendencies (*β* = 0.08, *b* = 0.98, 95% CI [-0.10, 2.07], *p* = 0.08), yet emotional reactivity suppressed the relationship between empathic disequilibrium and psychopathic tendencies (*β*_ab_ = -0.07, ab estimate = -0.83, 95% CI [-1.29, -0.46]). After controlling for emotional reactivity, an association emerged between empathic disequilibrium and psychopathic tendencies (*β* = 0.14, *b* = 1.82, 95% CI [0.72, 2.91], *p* = 0.001).

Total empathy was negatively correlated with psychopathic tendencies (*β* = -0.35, *b* = -2.68, 95% CI [-3.40, -1.97], *p* < 0.00001). Like empathic disequilibrium, emotional reactivity suppressed this association (*β*_ab_ = 0.11, ab estimate = 0.88, 95% CI [0.52, 1.28]), strengthening this association (*β* = -0.46, *b* = -3.56, 95% CI [-4.33, -2.80], *p* < 0.00001).

**Psychopathology**

Empathic disequilibrium was associated with depression (*β* = 0.06, *b* = 0.60, 95% CI [0.16, 1.04], *p* = 0.008), anxiety (*β* = -0.05, *b* = -0.48, 95% CI [-0.90, -0.05], *p* = 0.03) and interpersonal sensitivity (*β* = -0.09, *b* = -0.73, 95% CI [-1.24, -0.21], *p* = 0.006). No correlation was found with somatization (*β* = 0.04, *b* = 0.39, 95% CI [-0.27, 1.04], *p* = 0.25), hostility (*β* = 0.002, *b* = 0.02, 95% CI [-0.65, 0.69], *p* = 0.95), paranoid ideation (*β* = -0.01, *b* = -0.09, 95% CI [-0.67, 0.50], *p* = 0.77), phobic anxiety (*β* = -0.02, *b* = -0.20, 95% CI [-0.83, 0.44], *p* = 0.54), obsession compulsion (*β* = -0.01, *b* = -0.12, 95% CI [-0.64, 0.40], *p* = 0.65), and psychoticism (*β* = -0.01, *b* = -0.09, 95% CI [-0.57, 0.38], *p* = 0.70).

Total empathy was associated with anxiety (*β* = 0.07, *b* = 0.39, 95% CI [0.11, 0.76], *p* = 0.007), interpersonal sensitivity (*β* = 0.09, *b* = 0.47, 95% CI [0.13, 0.81], *p* = 0.007), paranoid ideation (*β* = -0.08, *b* = -0.43, 95% CI [-0.82, -0.50], *p* = 0.03), and phobic anxiety (*β* = 0.17, *b* = 0.86, 95% CI [0.44, 1.27], *p* = 0.00005). No association was found with depression (*β* = -0.03, *b* = -0.17, 95% CI [-0.46, 0.12], *p* = 0.25), somatization (*β* = -0.05, *b* = -0.30, 95% CI [-0.73, 0.13], *p* = 0.17), hostility (*β* = -0.08, *b* = -0.42, 95% CI [-0.86, 0.02], *p* = 0.06), obsession compulsion (*β* = 0.03, *b* = 0.17, 95% CI [-0.17, 0.51], *p* = 0.33), and psychoticism (*β* = 0.02, *b* = 0.09, 95% CI [-0.23, 0.40], *p* = 0.59).

**Depression**

While empathic disequilibrium was associated with depression (*β* = 0.06, *b* = 0.63, 95% CI [0.19, 1.08], *p* = 0.005), it was not mediated by emotional reactivity (*β*_ab_ = 0.008, ab estimate = 0.08, 95% CI [-0.05, 0.23]). Depressive symptoms were not associated with total empathy (*β* = -0.03, *b* = -0.21, 95% CI [-0.50, 0.09], *p* = 0.17), and this association was not mediated by emotional reactivity (*β*_ab_ = -0.02, ab estimate = -0.09, 95% CI [-0.23, 0.05]).

**Anxiety**

Empathic disequilibrium was associated with anxiety (*β* = -0.07, *b* = -0.60, 95% CI [-1.02, -0.17], *p* = 0.007). This association was fully mediated by emotional reactivity (*β*_ab_ = -0.04, ab estimate = -0.31, 95% CI [-0.48, -0.16]). After controlling for emotional reactivity, the association between empathic disequilibrium and anxiety became insignificant (*β* = -0.03, *b* = -0.29, 95% CI [-0.73, -0.15], *p* = 0.19).

Total empathy was associated with anxiety as well (*β* = 0.10, *b* = 0.50, 95% CI [0.22, 0.78], *p* = 0.0005). This association was fully mediated by emotional reactivity (*β*_ab_ = 0.06, ab estimate = 0.32, 95% CI [0.18, 0.48]), and became insignificant after controlling for emotional reactivity (*β* = 0.03, *b* = 0.18, 95% CI [-0.12, 0.49], *p* = 0.25).

**Interpersonal sensitivity**

Empathic disequilibrium towards EE-dominance was associated with interpersonal sensitivity (*β* = -0.09, *b* = -0.82, 95% CI [-1.34, -0.82], *p* = 0.002). This association was partially mediated by emotional reactivity (*β*_ab_ = -0.03, ab estimate = -0.24, 95% CI [-0.42, -0.08]) with emotional reactivity reducing the strength of this association (*β* = -0.07, *b* = -0.59, 95% CI [-1.12, -0.05], *p* = 0.03).

Total empathy was also associated with interpersonal sensitivity (*β* = 0.11, *b* = 0.57, 95% CI [0.23, 0.91], *p* = 0.001). Emotional reactivity fully mediated this association (*β*_ab_ = 0.05, ab estimate = 0.25, 95% CI [0.09, 0.43]), which became insignificant (*β* = 0.06, *b* = 0.32, 95% CI [-0.06, 0.69], *p* = 0.10).
